# The solid effect of dynamic nuclear polarization in liquids

**DOI:** 10.5194/mr-4-153-2023

**Published:** 2023-06-05

**Authors:** Deniz Sezer

**Affiliations:** Institute of Physical and Theoretical Chemistry, Goethe University, 60438 Frankfurt am Main, Germany

## Abstract

The solid-state effect of dynamic nuclear polarization (DNP) is operative also in viscous liquids where the dipolar interaction between the electronic and nuclear spins is partially averaged. The proper way to quantify the degree of averaging, and thus calculate the efficiency of the effect, should be based on the time-correlation function of the dipolar interaction. Here we use the stochastic Liouville equation formalism to develop a general theoretical description of the solid effect in liquids. The derived expressions can be used with different dipolar correlations functions depending on the assumed motional model. At high magnetic fields, the theory predicts DNP enhancements at small offsets, far from the classical solid-effect positions that are displaced by one nuclear Larmor frequency from the electronic resonance. The predictions are in quantitative agreement with such enhancement peaks observed at 9.4 T [Bibr bib1.bibx27]. These non-canonical peaks are not due to thermal mixing or the cross effect but exactly follow the dispersive component of the EPR line.

## 1 Introduction

The last 2 decades have witnessed an overarching development in nuclear hyperpolarization techniques across the entire spectrum of mechanisms, from the classical Overhauser and solid-state effects to (photo)chemically induced and parahydrogen-based polarization [Bibr bib1.bibx7]. While the majority of the reported applications have relied on polarization transfer in the solid state at cryogenic temperatures [Bibr bib1.bibx32], transfer in the liquid state at elevated (room or physiological) temperatures has also been actively explored [Bibr bib1.bibx36]. In the liquid state, the mechanism of polarization has almost exclusively been the Overhauser effect [Bibr bib1.bibx21]. One notable exception is the work of Stapf and coworkers in which the solid effect of dynamic nuclear polarization (DNP) has been employed in combination with field-cycling relaxometry to characterize the molecular dynamics in ionic liquids and polymer melts at ambient temperatures [Bibr bib1.bibx31].

At X band (9.6 GHz/0.35 T), where the DNP measurements of Stapf and colleagues have been carried out, the nuclear Larmor frequencies of ${}^{1}$ H and ${}^{19}$ F ( $\omega_{I}\approx 15$ MHz) are less than the EPR spectral width of a nitroxide free radical and even comparable to the spectral width of the single-line radical BDPA [Bibr bib1.bibx15]. As a result, the negative and positive solid-effect enhancements overlap and partly cancel each other, complicating the quantitative analysis of the effect. An additional difficulty for quantification is that, in many instances, the Overhauser and solid effects coexist [Bibr bib1.bibx29]. Although the contributions of these two effects can generally be distinguished on the basis of their even (Overhauser effect) and odd (solid effect) parity with the offset from the electronic resonance, this identification could be complicated when the EPR spectrum is broad and asymmetrical.

To quantify the field profile of the DNP enhancement (i.e., the DNP spectrum), Stapf and colleagues use a weighted sum of (i) the EPR line shape (for the Overhauser effect) and (ii) the same line shape shifted by $\pm \omega_{I}$ , with one of the shifted copies flipped around the vertical axis (for the solid effect) [Bibr bib1.bibx31]. The relative contribution of the two effects is then treated as a fitting parameter. In general, the resulting fits are in good overall agreement with the experimental DNP spectra, but oftentimes there are quantitative deviations. Recently, the remaining discrepancy between the experimental and calculated DNP spectra was interpreted as evidence for the simultaneous occurrence of a third DNP mechanism, in addition to the Overhauser and solid effects [Bibr bib1.bibx16].

In the companion paper [Bibr bib1.bibx37] we showed that when the microwave (mw) nutation frequency approaches the nuclear Larmor frequency, as could be the case at X band under the high mw powers used in the DNP experiments [Bibr bib1.bibx30], the forbidden transitions of the solid effect are no longer shifted by $\pm \omega_{I}$ with respect to the electronic resonance but come closer together and may even coalesce. In such cases, the theoretical justification for modeling the solid-effect field profile by shifting the EPR line shape by $\pm \omega_{I}$ becomes questionable. Unfortunately, the analytical expressions of [Bibr bib1.bibx37], which remain valid in this regime, are not applicable to liquids since they do not account for the modulation of the dipolar interaction by molecular diffusion. In the current paper, the time-domain description of the solid effect from [Bibr bib1.bibx37] is extended to liquids.

Recently, [Bibr bib1.bibx27] reported proton DNP enhancements in the liquid phase of lipid bilayers at 9.4 T (260 GHz) using the free radical BDPA as a polarizing agent. The large nuclear Larmor frequency (400 MHz) enabled a clear spectral separation of the acyl-chain protons and the polar protons of water. For the non-polar protons, maximal enhancements were observed at the canonical resonance positions of the zero- and double-quantum forbidden transitions, characteristic of the solid effect, while enhancement due to the Overhauser effect was missing. The large spectral separation of the positive and negative enhancements and the narrow line of BDPA provided a uniquely “clean” access to the solid effect in a viscous liquid environment [Bibr bib1.bibx27].

In addition to the maximum enhancements at the canonical solid-effect offsets, the DNP spectrum of [Bibr bib1.bibx27] revealed additional enhancement peaks at much smaller offsets. These were postulated to arise due to the DNP mechanism known as thermal mixing. However, thermal mixing is commonly associated with a broad EPR spectrum [Bibr bib1.bibx43], while the spectrum of BDPA was extremely narrow at the elevated DNP concentration used in the experiment, and the observed spurious enhancement peaks lay outside this narrow spectrum Fig. 2. Here we explain the entire DNP spectrum, including the puzzling features at low offsets, considering only one electronic and one nuclear spin.

The rest of the paper is organized as follows. To account for molecular diffusion, in Sect. [Sec Ch1.S3] we transform the equations of motion of [Bibr bib1.bibx37] into stochastic Liouville equations (SLEs) [Bibr bib1.bibx25]. After taking into account that all relevant timescales are orders of magnitude shorter than the nuclear spin-lattice relaxation time, the SLE formalism yields the time-correlation function of the dipolar interaction. In Sect. [Sec Ch1.S4] we show that the solid-effect lines in the DNP spectrum (i.e., those shifted by $\pm \omega_{I}$ ) experience additional motional broadening compared to the homogeneous EPR line width. As a result, the tails of these lines around the position of the electronic resonance increase substantially. Under favorable conditions, the product of these tails with the dispersive EPR component may become sufficiently large to be visible as separate enhancement peaks in the DNP field profile. We attribute the non-canonical peaks in the DNP spectrum of [Bibr bib1.bibx27] to this phenomenon. Our conclusions are presented in Sect. [Sec Ch1.S5]. The next section summarizes the needed background.

## 2 Motivation and background

### 2.1 Dynamic nuclear polarization in liquids

The transfer of polarization in solids involves two mechanistically different steps [Bibr bib1.bibx19]. The first one is the direct polarization of the nuclear spins that are sufficiently close to the free radical and have appreciable dipole–dipole interaction with the electronic spin. Being closest to the unpaired electron, the nuclei on the free radical itself benefit most from this first step of direct polarization [Bibr bib1.bibx40]. Polarizing the intramolecular nuclei in this way, however, is not particularly useful unless the polarization can spread to the rest of the sample. This is where the second step comes in. In this step, the polarization spreads from the directly polarized nuclei to the distant nuclei by spin diffusion. Because it relies on the relatively weak dipole–dipole interaction between the nuclear spins, spin diffusion is slow and is often the bottleneck for efficient polarization transfer in the solid state [Bibr bib1.bibx19].

In liquids, spin diffusion is not efficient because the nuclei constantly change their positions due to random thermal motions. However, since molecular diffusion moves the nuclei across nanometer distances in nanoseconds and thus rapidly spreads the polarization of the directly polarized nuclei across the sample, spin diffusion is also not needed. Taking glycerol as an example, with a self-diffusion coefficient of $6.6\times 10^{-3}$ nm ${}^{2}$ ns ${}^{-1}$ at 40 ${}^{\circ }$ C [Bibr bib1.bibx41], which is 500 times less than the self-diffusion coefficient of water at the same temperature [Bibr bib1.bibx18], it is a rather viscous liquid. Nevertheless, at the relatively small radical concentration of 1 mM, a molecule of glycerol covers the average distance between two radicals in less than 400 ns. This is at least 5 orders of magnitude less than the nuclear $T_{1}$ of protons, even after accounting for paramagnetic relaxation. Any given solvent nucleus will thus encounter the electronic spins a million times during its $T_{1}$ relaxation time. Even in viscous liquids, therefore, molecular diffusion is expected to homogenize the nuclear polarization across the sample during times that are orders of magnitude shorter than the nuclear $T_{1}$ . This advantage of liquids over solids, however, comes at a price: the polarization of the nuclei on the free radical is no longer accessible to the solvent, and proximal solvent nuclei have to be polarized directly in the first step of polarization transfer (Fig. [Fig Ch1.F1]).

**Figure 1 Ch1.F1:**
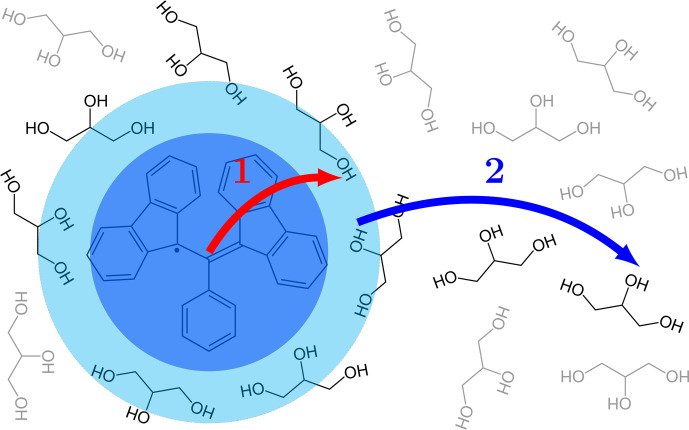
Two conceptually different steps of the polarization transfer process in liquids. (1) Direct transfer from the electronic spin on the free radical to the proximate nuclear spins on the solvent molecules due to dipolar interaction. (2) Diffusion of the proximate solvent molecules to the bulk.

Given that every solvent molecule gets directly polarized and also spreads the polarization, the distinction between the two steps of polarization transfer in liquids (Fig. [Fig Ch1.F1]) is conceptual and does not reflect fundamental differences in the mechanisms of the two steps. In fact, both steps are enabled by molecular diffusion which sometimes brings a solvent molecule closer to the radical and sometimes takes it further away. Since the analytical description of translational diffusion in simple liquids is well developed [Bibr bib1.bibx4], a unified theoretical treatment of the two steps of polarization transfer becomes possible, as we demonstrate in the present paper.

From the six terms of the dipolar alphabet, the part that contributes to the solid effect is $A_{1}S_{z}I_{+}+A_{1}^{*}S_{z}I_{-}$ [Bibr bib1.bibx42], where

1 $$A_{1}=D_{\mathrm{dip}}\frac{-3\mathrm{cos}\theta \mathrm{sin}\theta e^{-i\phi }}{r^{3}}.$$

Here $D_{\mathrm{dip}}=\left(\mu_{0}/4\pi \right)\hbar \gamma_{S}\gamma_{I}$ is the dipolar constant, $\gamma_{S}$ and $\gamma_{I}$ are the gyromagnetic ratios of the electronic and nuclear spins, and (r,θ,ϕ) are the spherical polar coordinates of the vector pointing from one of the spins to the other. The angular dependence of $A_{1}$ is that of a second-degree spherical harmonic of order m=1 , as implied by the subscript. The need for direct polarization of the solvent nuclei in liquids increases the shortest possible distance r in Eq. ([Disp-formula Ch1.E1]) and thus reduces the largest achievable dipolar coupling. This requirement for interaction across a larger distance, however, does not explain why the solid effect works in solids but is compromised in liquids.

To understand the difference between solids and liquids one should consider the time-correlation function of the dipolar interaction:

2 $$C_{11}(t)=\langle \langle A_{1}^{*}(t^{\prime }+t)A_{1}(t^{\prime })\rangle_{t^{\prime }}\rangle .$$

Here the inner angular brackets with the subscript $t^{\prime }$ denote averaging with respect to the time point $t^{\prime }$ along the random trajectory of a single nuclear spin. Because every nucleus encounters the electronic spins millions of times during its $T_{1}$ relaxation time, this average should be the same for all nuclei in the liquid. Thus, in addition to the time averaging, in Eq. ([Disp-formula Ch1.E2]) we also average over the ensemble of identical nuclear spins in the sample (outer angular brackets).

Now, if the dipolar correlation function (Eq. [Disp-formula Ch1.E2]) decays on timescales that are much longer than some relevant characteristic time, then the experiment essentially detects the initial value $C_{11}(0)=\langle \langle A_{1}^{*}(t^{\prime })A_{1}(t^{\prime })\rangle_{t^{\prime }}\rangle =\langle A_{1}^{*}A_{1}\rangle$ . The last ensemble average over all electron–nucleus pairs requires integration over the spatial variables (r,θ,ϕ) and multiplication by the concentration N of the unpaired electrons:

3 $$\langle A_{1}^{*}A_{1}\rangle =ND_{\mathrm{dip}}^{2}\frac{24\pi }{5}\int_{b}^{\infty }\frac{dr}{r^{4}}=D_{\mathrm{dip}}^{2}\frac{24\pi }{5}\frac{N}{3b^{3}}.$$

(The factor 24π/5 comes from the normalization of the spherical harmonic $Y_{2}^{1}$ .) This slow-motional limit corresponds to the situation in solids under the (unrealistic) assumption of fast and efficient spin diffusion. If, on the other hand, the decay time of the correlation function is much shorter than the relevant characteristic timescale, then the experiment detects the long-time limit $C_{11}(\infty )=\langle \langle A_{1}^{*}(\infty )A_{1}(t^{\prime })\rangle_{t^{\prime }}\rangle =\langle A_{1}^{*}\rangle \langle A_{1}\rangle$ . The solid effect vanishes because the average of the spherical harmonic $Y_{2}^{1}$ over the angles gives $\langle A_{1}\rangle =0$ . This fast-motional limit corresponds to low-viscosity liquids in which the dipolar interaction is averaged out. To the extent that they exhibit the solid effect, viscous liquids must lie somewhere between these two extremes.

The interpolation between these two limiting cases on the basis of the dipolar correlation function is formally developed in Sect. [Sec Ch1.S3]. This task requires a time-domain description of the solid effect, similar to the treatment of relaxation by random motion where the correlation function arises from second-order, time-dependent perturbation theory ([Bibr bib1.bibx1], chap. VIII). In principle, there are two such time-domain descriptions that we can utilize for the treatment of the solid effect in liquids. The first is the rate-equation formalism, which models the dynamics of the electronic and nuclear polarizations, and the second is the description developed in [Bibr bib1.bibx37], which additionally accounts for the dynamics of the coherences. Both of these options will be explored in Sect. [Sec Ch1.S3]. When modeling the stochastic dynamics of the dipolar interaction, we resort to the stochastic Liouville equation (SLE) of [Bibr bib1.bibx24] and [Bibr bib1.bibx3], rather than to second-order perturbation theory. In agreement with previous work [Bibr bib1.bibx34], our analysis shows that the characteristic timescale against which the dipolar correlation time should be compared is the electronic $T_{2}$ relaxation time.

In the next two subsections we summarize the time-domain analysis of [Bibr bib1.bibx37].

### 2.2 Rate equations

The dynamics of the electronic polarization, $P_{S}$ , is justifiably taken to be independent of the dipolar interaction with the nuclear spins, as other mechanisms relax the electrons more efficiently. With $R_{1S}$ denoting the rate of electronic $T_{1}$ relaxation, and $v_{1}$ the rate constant of the mw excitation of the (allowed) EPR transition, the rate equation of the electronic polarization is

4 $${\dot{P}}_{S}=-R_{1S}(P_{S}-P_{S}^{\mathrm{eq}})-2v_{1}P_{S}.$$

Here $P_{S}^{\mathrm{eq}}$ is the equilibrium (Boltzmann) electronic polarization and the dot over the symbol denotes differentiation with respect to time. Solving this equation for $P_{S}^{\mathrm{ss}}$ at steady state, we arrive at the ratio

5 $$p=\frac{P_{S}^{\mathrm{ss}}}{P_{S}^{\mathrm{eq}}}=\frac{R_{1S}}{R_{1S}+2v_{1}}=1-s,$$

where s is the familiar electronic saturation factor. We refer to p as the electronic polarization factor, since p=0 indicates that the steady-state polarization has vanished, and p=1 indicates that it is identical to the Boltzmann polarization (i.e., maximally polarized).

The rate equation of the nuclear polarization, $P_{I}$ , is

6 $${\dot{P}}_{I}=-R_{1I}\left(P_{I}-P_{I}^{\mathrm{eq}}\right)-v_{+}P_{I}-v_{-}P_{S},$$

where $R_{1I}$ is the nuclear $T_{1}$ relaxation rate, and the phenomenological rate constants $v_{\pm }$ quantify the mw excitation of the forbidden transitions. The steady state of Eq. ([Disp-formula Ch1.E6]) is

7 $$R_{1I}\left(P_{I}^{\mathrm{ss}}-P_{I}^{\mathrm{eq}}\right)=-v_{+}P_{I}^{\mathrm{ss}}-pv_{-}P_{S}^{\mathrm{eq}},$$

where $P_{S}^{\mathrm{ss}}=pP_{S}^{\mathrm{eq}}$ was used in the last term.

As the derivations in Sect. [Sec Ch1.S3] consider only the effect of mw excitation, we have written Eq. ([Disp-formula Ch1.E7]) such that the relaxation contribution is on the left and the mw contribution on the right of the equality. Subsequently, to identify the phenomenological rate constants $v_{\pm }$ , we will match the terms on the right-hand side with the predictions of the proper analysis in liquids.

From Eq. ([Disp-formula Ch1.E7]) we find the DNP enhancement

8 $$\epsilon =P_{I}^{\mathrm{ss}}/P_{I}^{\mathrm{eq}}-1=\epsilon_{\mathrm{SE}}-\left(1-p_{X}\right),$$

where the first equality is the definition of ϵ and

9 $$\epsilon_{\mathrm{SE}}=\frac{pv_{-}}{R_{1I}+v_{+}}\frac{\left|\gamma_{S}\right|}{\gamma_{I}},\;p_{X}=\frac{R_{1I}}{R_{1I}+v_{+}}.$$

From $\epsilon_{\mathrm{SE}}$ it is clear that the solid effect benefits from large $pv_{-}$ and small $R_{1I}+v_{+}$ . The ratio $p_{X}$ in Eq. ([Disp-formula Ch1.E9]) is analogous to the electronic polarization factor Eq. ([Disp-formula Ch1.E5]), and we call it the nuclear cross-polarization factor. In liquids, $v_{+}$ is typically negligible compared to the nuclear spin-lattice relaxation rate, and $p_{X}\approx 1$ . Then,

10 $$\epsilon_{\mathrm{SE}}\approx (pv_{-})T_{1I}|\gamma_{S}|/\gamma_{I}\;(p_{X}\approx 1).$$

### 2.3 Spin dynamics

The dynamics of the quantum-mechanical expectation values $s_{n}$ of the electronic spin operators $S_{n}$ ( n=x,y,z ) is described by the classical Bloch equations

11 $$\left[\begin{array}{c} {\dot{s}}_{x}\\ {\dot{s}}_{y}\\ {\dot{s}}_{z} \end{array}\right]=-\left[\begin{array}{ccc} R_{2S} & \Omega & 0\\ -\Omega & R_{2S} & \omega_{1}\\ 0 & -\omega_{1} & R_{1S} \end{array}\right]\left[\begin{array}{c} s_{x}\\ s_{y}\\ s_{z} \end{array}\right]+R_{1S}\left[\begin{array}{c} 0\\ 0\\ s_{z}^{\mathrm{eq}} \end{array}\right].$$

The matrix in Eq. ([Disp-formula Ch1.E11]) contains the electronic transverse relaxation rate $R_{2S}$ , the mw nutation frequency $\omega_{1}$ and the offset $\Omega =\omega_{S}-\omega$ between the electronic Larmor frequency $\omega_{S}$ and the mw frequency ω .

In [Bibr bib1.bibx37] we visualized such coupled differential equations diagrammatically. In our visual depiction, the time derivative of a dynamical variable, like $s_{n}$ , is represented by an oval node. The contributions to this time derivative, which are on the right-hand side of the differential equation, are represented by arrows that flow into that node (Fig. [Fig Ch1.F2]a). The contribution of a given arrow is obtained by multiplying the weight of the arrow by the variable from which the arrow originates. The self-arrows that exit from an oval node and enter the same node correspond to the relaxation terms along the diagonal of the Bloch matrix. The negative sign of the weight of a self-arrow is written separately inside the loop formed by the arrow. The constant variable $s_{z}^{\mathrm{eq}}$ in the inhomogeneous term of the Bloch equations (Eq. [Disp-formula Ch1.E11]) is represented by a gray rectangular node. With this notation, the Bloch equations are depicted by the four nodes in the top row of Fig. [Fig Ch1.F2]a and by the black arrows connecting these nodes.

**Figure 2 Ch1.F2:**
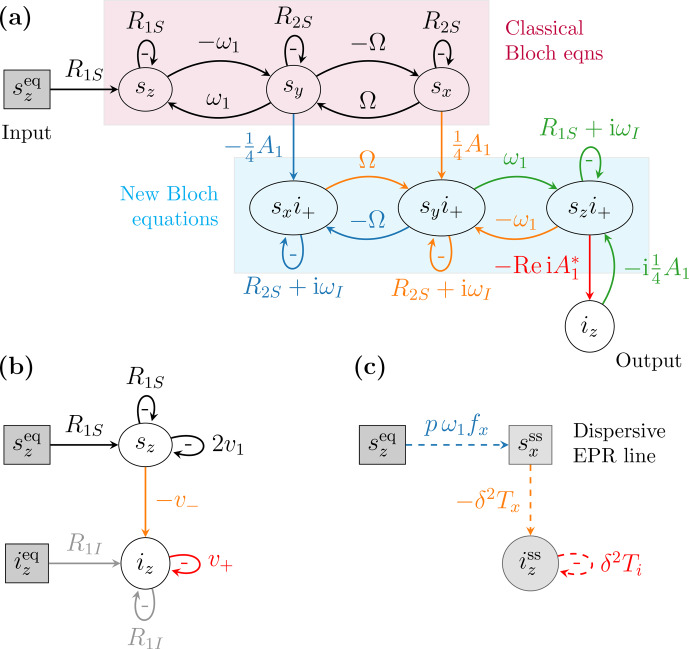
Diagrammatic representation of **(a)** the equations of motion of the spin dynamics relevant to the solid effect, namely Eq. ([Disp-formula Ch1.E11]) (purple rectangle), Eq. ([Disp-formula Ch1.E12]) (cyan rectangle) and Eq. ([Disp-formula Ch1.E14]), and **(b)** the rate equations of the electronic and nuclear polarizations (Eqs. [Disp-formula Ch1.E4] and [Disp-formula Ch1.E6]). **(c)** Steady-state relationships between the input and output variables.

The lower half of Fig. [Fig Ch1.F2]a shows the dynamics of the electron–nuclear coherences that are relevant for the solid effect. In particular, the quantum-mechanical expectation values of the operators $S_{n}I_{+}$ ( n=x,y,z ), which we denote interchangeably by $g_{n}$ and $s_{n}i_{+}$ , evolve according to the following coupled differential equations [Bibr bib1.bibx37]:

12 $$\left[\begin{array}{c} {\dot{g}}_{x}\\ {\dot{g}}_{y}\\ {\dot{g}}_{z} \end{array}\right]=-B\left[\begin{array}{c} g_{x}\\ g_{y}\\ g_{z} \end{array}\right]-i\frac{1}{4}A_{1}\left[\begin{array}{c} -is_{y}\\ is_{x}\\ i_{z} \end{array}\right],$$

where

13 $$B=\left[\begin{array}{ccc} R_{2S}+i\omega_{I} & \Omega & 0\\ -\Omega & R_{2S}+i\omega_{I} & \omega_{1}\\ 0 & -\omega_{1} & R_{1S}+i\omega_{I} \end{array}\right].$$

The matrix B is essentially the Bloch matrix but with the nuclear Larmor frequency $\omega_{I}$ added as an imaginary part to its main diagonal. The time derivatives of $g_{n}$ in Eq. ([Disp-formula Ch1.E12]) are represented by the three oval nodes enclosed in the cyan rectangle in Fig. [Fig Ch1.F2]a. The arrows between these nodes are seen to exactly replicate the classical Bloch equations in the rectangle above them. The inhomogeneous term in Eq. ([Disp-formula Ch1.E12]) couples the dynamics of the variables $g_{n}$ to the transverse components of the electronic magnetization, on the one hand, and to the longitudinal component of the nuclear magnetization, on the other. All these couplings scale with the dipolar interaction $A_{1}$ . They play an essential role in the solid effect, as they connect the Boltzmann electronic polarization to the nuclear polarization (labeled “Input” and “Output” in Fig. [Fig Ch1.F2]a).

Lastly, the coherent dynamics of the operator $I_{z}$ , whose expectation value is denoted by $i_{z}$ , is

14 i˙z|coh=-Re{iA1*gz},

where Re takes the real part of a complex number. In Fig. [Fig Ch1.F2]a this equation is represented by the oval node $i_{z}$ (labeled “Output”) and the red arrow flowing into it.

In liquids, the weights $A_{1}$ fluctuate randomly due to molecular diffusion. When extending the formalism to liquids (Sect. [Sec Ch1.S3.SS3]), we will transform Eq. ([Disp-formula Ch1.E12]), which constitutes a system of coupled differential equations, to an SLE [Bibr bib1.bibx25] that describes the spin dynamics under random modulation of $A_{1}$ .

For comparison, Fig. [Fig Ch1.F2]b shows the dynamics of the longitudinal components implied by the rate equations (Eqs. [Disp-formula Ch1.E4] and [Disp-formula Ch1.E6]). Visual inspection of Fig. [Fig Ch1.F2]a and b makes it clear that the rate constant $v_{-}$ provides a reduced description of the complicated network connecting $s_{z}$ to $i_{z}$ . Similarly, the rate constant $v_{+}$ accounts for the self-influence of $i_{z}$ mediated by the coherences in the second set of Bloch equations (enclosed in the cyan rectangle). In [Bibr bib1.bibx37], we identified the rates $v_{\pm }$ and $v_{1}$ by requiring that the dynamics in Fig. [Fig Ch1.F2]a and b reached identical steady states.

At steady state, the three dynamical variables of the classical Bloch equations (Eq. [Disp-formula Ch1.E11]) were related to each other and to the electronic Boltzmann polarization as follows:

15 $$s_{x}^{\mathrm{ss}}=\left(\omega_{1}f_{x}\right)s_{z}^{\mathrm{ss}},\;s_{y}^{\mathrm{ss}}=-\left(\omega_{1}f_{y}\right)s_{z}^{\mathrm{ss}},\;s_{z}^{\mathrm{ss}}=\left(R_{1S}f_{z}\right)s_{z}^{\mathrm{eq}},$$

where

16 $$f_{y}=\frac{R_{2S}}{R_{2S}^{2}+\Omega^{2}},\;\;f_{x}=\frac{\Omega }{R_{2S}}f_{y},\;\;f_{z}=\frac{1}{R_{1S}+\omega_{1}^{2}f_{y}}.$$

(Comparing the last equality in Eq. ([Disp-formula Ch1.E15]) with Eq. ([Disp-formula Ch1.E5]) we found that $p=R_{1S}f_{z}$ and that the rate constant of the allowed EPR transition was $v_{1}=\omega_{1}^{2}f_{y}/2$ .)

Solving the second set of Bloch equations (Eq. [Disp-formula Ch1.E12]) at steady state, and substituting $g_{z}^{\mathrm{ss}}$ in Eq. ([Disp-formula Ch1.E14]), we obtain

17 i˙z|cohss=-δ2Re001B-1-isyssisxssizss,

where the dipolar interaction is isolated in

18 $$\delta^{2}=\left(A_{1}^{*}A_{1}\right)/4.$$

Since the transverse components $s_{x,y}^{\mathrm{ss}}$ are related to the Boltzmann polarization (Eq. [Disp-formula Ch1.E15]), the right-hand side of Eq. ([Disp-formula Ch1.E17]) is of the form

19 $${\dot{i}}_{z}{|}_{\mathrm{coh}}^{\mathrm{ss}}=-\delta^{2}\left(T_{i}i_{z}^{\mathrm{ss}}+T_{s}s_{z}^{\mathrm{eq}}\right),$$

where

20 Ti=001Re{B-1}001T=Re{B33-1}Ts=001Re{iB-1}pω1fypω1fx0T.

(The superscript “T” indicates transpose, and $B_{ij}^{-1}$ is the ij th element of the inverse matrix $B^{-1}$ .) Because B has units of inverse time, $T_{i}$ and $T_{s}$ have units of time.

We note that $T_{s}$ receives contributions from both $s_{y}^{\mathrm{ss}}$ and $s_{x}^{\mathrm{ss}}$ . As was shown in [Bibr bib1.bibx37], it is possible to rewrite the contribution of the former as if it also came from $s_{x}^{\mathrm{ss}}$ . In other words, the entire contribution of $s_{z}^{\mathrm{eq}}$ to the derivative of $i_{z}$ at steady state can be expressed as if it is mediated only through the dispersive component $s_{x}^{\mathrm{ss}}$ , as depicted in Fig. [Fig Ch1.F2]c. (Dashed arrows represent mathematical relationships between the variables that hold at steady state. Differently from the solid arrows, which correspond to causal dependencies governing the dynamics at all times, the dashed arrows need not reflect direct causal dependence. A rectangular node indicates that the inflowing arrows contribute directly to the value of the variable and not to its time derivative. The gray shade of the nodes signals that the variables remain constant in time, as they should at steady state.)

In addition to $B_{33}^{-1}$ , in Eq. ([Disp-formula Ch1.E20]) we also need $B_{31}^{-1}$ and $B_{32}^{-1}$ . These are $B_{31}^{-1}=(\omega_{1}F_{x})F_{z}$ , $B_{32}^{-1}=(\omega_{1}F_{y})F_{z}$ and $B_{33}^{-1}=F_{z}$ , where the functions

21 $$\begin{array}{rl} F_{y} & =\frac{R_{2S}+i\omega_{I}}{{\left(R_{2S}+i\omega_{I}\right)}^{2}+\Omega^{2}},\;F_{x}=\frac{\Omega }{R_{2S}+i\omega_{I}}F_{y}\\ F_{z} & =\frac{1}{R_{1S}+i\omega_{I}+\omega_{1}^{2}F_{y}} \end{array}$$

play an analogous role in the steady-state analysis of the second set of Bloch equations as the functions $f_{y}$ , $f_{x}$ and $f_{z}$ (Eq. [Disp-formula Ch1.E16]) in the classical Bloch equations. In terms of these,

22 Ti=Re{Fz},Ts=pω1fxTx,

where

23 Tx=Re{iFz(ω1Fy′)},Fy′=2R2S+iωIR2S+iωIFy.

(The functions in Eq. [Disp-formula Ch1.E23] emerge from lumping the contribution of $s_{y}^{\mathrm{ss}}$ to that of $s_{x}^{\mathrm{ss}}$ , as mentioned above.)

With $T_{i}$ and $T_{s}$ determined, the forbidden-transition rates on the right-hand side of Eq. ([Disp-formula Ch1.E7]) become

24 $$v_{+}=\delta^{2}\left(T_{i}-T_{i}^{0}\right),\;pv_{-}=\delta^{2}T_{s}=\left(p\omega_{1}f_{x}\right)\delta^{2}T_{x},$$

where the mw-independent part of $T_{i}$ , namely

25 Ti0=ReFzω1=0=R1S+iωI-1,

is subtracted in the first equality of Eq. ([Disp-formula Ch1.E24]) because it contributes to the nuclear $T_{1}$ relaxation rate. (In Fig. [Fig Ch1.F2]a this mw-independent part corresponds to the loop formed by the green arrow from $i_{z}$ to $g_{z}$ and the red arrow in the opposite direction.)

### 2.4 The solid effect

Using the rate constants in Eq. ([Disp-formula Ch1.E24]), we rewrite the solid-effect DNP enhancement (Eq. [Disp-formula Ch1.E9]) as

26 $$\epsilon_{\mathrm{SE}}=\frac{\left(p\omega_{1}f_{x}\right)T_{x}}{R_{1I}/\delta^{2}+\left(T_{i}-T_{i}^{0}\right)}\frac{\left|\gamma_{S}\right|}{\gamma_{I}}.$$

The functions $p\omega_{1}f_{x}$ , $T_{x}$ and $\left(T_{i}-T_{i}^{0}\right)$ in this expression are visualized in, respectively, the first, second and third rows of Fig. [Fig Ch1.F3]. The product of the first two rows, which appears in the numerator of Eq. ([Disp-formula Ch1.E26]), is shown in the fourth row of the figure. In the right margin of the figure, we have included the flow diagram from Fig. [Fig Ch1.F2]c, which has been straightened here so that the weights of the arrows correspond to the respective rows. Note that the dipolar interaction strength, δ , and the nuclear spin-lattice relaxation rate were not needed to calculate the properties in the first four rows of the figure. (They will be needed for the last two rows.)

**Figure 3 Ch1.F3:**
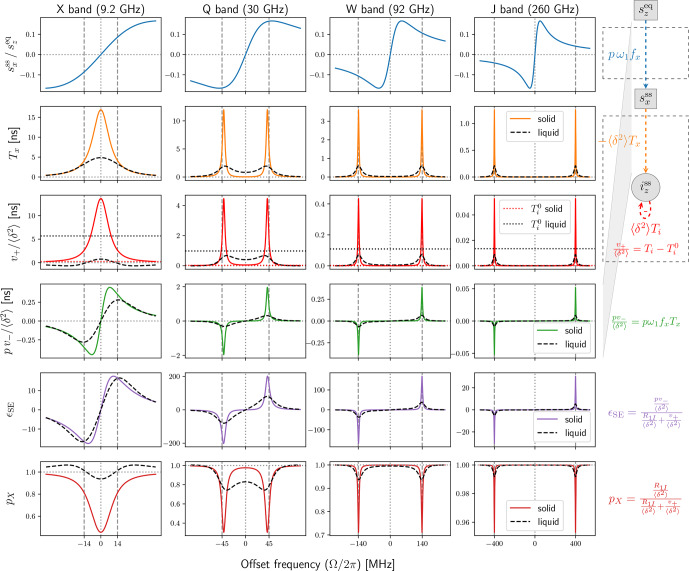
Comparison between solids (with fast spin diffusion) and liquids. First row: dispersive component of the power-broadened EPR line. Second and third rows: relevant transfer functions of the new Bloch equations in Fig. [Fig Ch1.F2]a. Fourth row: the product of the first and second rows, which relates the input $s_{z}^{\mathrm{eq}}$ to the output $i_{z}^{\mathrm{ss}}$ . Fifth row: DNP enhancement calculated from the third and fourth rows using $R_{1I}/\langle \delta^{2}\rangle$ . Sixth row: nuclear cross-polarization factor calculated from the third row using $R_{1I}/\langle \delta^{2}\rangle$ . Simulation parameters: $T_{2S}=60$ ns, $T_{1S}=9\;T_{2S}$ , $B_{1}=6$ G (converted to $\omega_{1}$ assuming g=2 ), contact distance b=1 nm, radical concentration N=0.1 M, and $T_{1I}$ of 4.7 ms (X band), 27.4 ms (Q band) and 50 ms (W and J bands). The dipolar correlation time of the liquid simulation (dashed black lines) is $\tau =T_{2S}/5=12$ ns.

While different magnetic fields $B_{0}$ yield different relaxation times, for illustrative purposes we used the same electronic $T_{1}$ and $T_{2}$ times for X, Q, W and J bands. We additionally used the same mw field ( $B_{1}=6$ G) at all bands. Hence, the steady state of the classical Bloch equations (Fig. [Fig Ch1.F3], first row) is identical across the four columns of the figure. The solid blue lines, which correspond to the dispersive component of the power-broadened EPR line, are identical but appear different due to the different scalings of the horizontal axes. The absorptive component is much smaller under the power-broadening conditions considered here and is not shown. However, its contribution is exactly accounted for in the analysis. (This was the reason for introducing the functions in Eq. [Disp-formula Ch1.E23].) Anticipating the liquid state, we observe that the classical Bloch equations are independent of the dipolar coupling $A_{1}$ (Fig. [Fig Ch1.F2]a). Hence, the first row of Fig. [Fig Ch1.F3] will not change when going to liquids because we use identical relaxation rates.

The transfer functions $T_{x}$ and $\left(T_{i}-T_{i}^{0}\right)$ encapsulate all relevant steady-state properties of the second set of Bloch equations, as well as their coupling to the classical Bloch equations and to $i_{z}$ through the dipolar interaction (Fig. [Fig Ch1.F2]a). These functions are visualized in the second and third rows of Fig. [Fig Ch1.F3] (orange and red lines). The solid colored lines are calculated using the equations given above and correspond to solids, subject to the (unrealistic) assumption of very fast spin diffusion. The dashed black lines are calculated as described in the next section and correspond to liquids. Clearly, the time-dependent modulation of the dipolar interaction in liquids has a dramatic effect on these functions.

The fourth row in Fig. [Fig Ch1.F3] shows the product of the blue lines in the first row and the orange lines in the second row and corresponds to the total transfer function from the primary input, $s_{z}^{\mathrm{eq}}$ , to the ultimate output, $i_{z}$ (Fig. [Fig Ch1.F2]a). With the exception of X band, going from solids to liquids substantially reduces the peaks of $pv_{-}$ . (We used identical relaxation parameters for liquids and solids to highlight the role of the dipolar correlation time.)

The transfer functions in the first four rows of Fig. [Fig Ch1.F3] depend only on the electronic relaxation times (assuming $T_{2I}\gg T_{1S},T_{2S}$ ). To calculate the enhancement $\epsilon_{\mathrm{SE}}$ and the nuclear cross-polarization factor, $p_{X}$ , which are shown in the last two rows of Fig. [Fig Ch1.F3], we had to select specific values for $R_{1I}$ and $\delta^{2}$ . For the latter, we used the ensemble-averaged static value from Eq. ([Disp-formula Ch1.E3]),

27 $$\langle \delta^{2}\rangle =\frac{1}{4}\langle A_{1}^{*}A_{1}\rangle =D_{\mathrm{dip}}^{2}\frac{6\pi }{5}\frac{N}{3b^{3}},$$

which applies to solids with fast and efficient spin diffusion. The numerical calculations in Fig. [Fig Ch1.F3] are for contact distance $b_{\mathrm{ref}}=1$ nm and radical concentration $N_{\mathrm{ref}}=0.1$ M. These are realistic but otherwise arbitrary values.

For the purposes of illustration we wanted to use the same $R_{1I}$ for all four mw bands in the figure. In this way, by comparing the four columns with each other, one would be able to assess the effect of changing only $B_{0}$ . This strategy worked for solids, at least for the numerical values that were used, but failed for liquids due to the very different contributions of $T_{i}$ to the nuclear relaxation rate (denoted by $T_{i}^{0}$ in Eq. [Disp-formula Ch1.E25]). This part of $T_{i}$ is shown in the third row of Fig. [Fig Ch1.F3] with horizontal dotted lines. The dotted red lines for solids are very close to zero. The more visible dotted black lines for liquids change dramatically with the mw band. Since the total relaxation rate $R_{1I}$ must be larger than the contribution of $T_{i}^{0}$ , the nuclear $T_{1}$ had to be only a few ms at X band. Using such small $T_{1}$ at J band, however, gave tiny liquid-state DNP enhancements.

Even if, admittedly, our calculated enhancements are only illustrative, in an effort to have somewhat realistic nuclear $T_{1}$ relaxation times, we used $T_{1I}=50$ ms when the corresponding rate $R_{1I}$ was larger than $\langle \delta^{2}\rangle T_{i}^{0}$ and used $R_{1I}/\langle \delta^{2}\rangle =2T_{i}^{0}$ otherwise. This resulted in the following nuclear $T_{1}$ relaxation times: 4.7 ms (X band), 27.4 ms (Q band) and 50 ms (W and J bands). These were used for both liquids and solids. Naturally, the choice of different nuclear $T_{1}$ times has a direct influence on the calculated enhancements. For example, the peak DNP enhancements at X and W bands differ by about 1 order of magnitude (purple lines in the fifth row of Fig. [Fig Ch1.F3]) mostly because the nuclear $T_{1}$ times at these two bands also differ by 1 order of magnitude.

The theory behind the liquid calculations in Fig. [Fig Ch1.F3] is presented in the next section.

## 3 Liquids

### 3.1 Molecular motion as a random process

Let us denote the components (r,θ,ϕ) of the inter-spin vector collectively by ζ . To describe the solid effect in liquids we consider a random process that changes ζ and thus modulates the dipolar interaction between the two types of spins.

When the random dynamics of ζ is modeled as a discrete-state process, the probabilities of observing the different discrete states are collected in the vector p(t) . This probability vector evolves in time as $\dot{p}(t)=-Kp(t)$ , where the matrix K contains the rate constants of the random transitions between the states. All eigenvalues of such stochastic matrices are non-negative, and, for an ergodic chain of states, only one of the eigenvalues equals zero. In general, the stochastic matrix K is not symmetric, which means that there are a right eigenvector and a left eigenvector associated with each eigenvalue. The right eigenvector of the zero eigenvalue corresponds to the vector of equilibrium probabilities, $p^{\mathrm{eq}}$ , and the left eigenvector of the zero eigenvalue corresponds to the vector 1 , which contains ones in all of its entries. Note that $1^{T}p^{\mathrm{eq}}=1$ .

When the random dynamics of ζ is modeled as a continuous-state diffusion process, then the time evolution of the probability density p(ζ,t) is described by a Fokker–Planck equation of the form

28 $$\frac{\partial p(\zeta ,t)}{\partial t}=-K_{\zeta }p(\zeta ,t),$$

where $K_{\zeta }$ is a linear differential operator acting on the ζ dependence of p(ζ,t) . As in the discrete case, the eigenvalues of $K_{\zeta }$ would be non-negative, and one eigenvalue would equal zero. The corresponding right eigenfunction is the equilibrium probability density $p^{\mathrm{eq}}(\zeta )$ , and the left eigenfunction is constant in ζ .

For brevity, we will also adopt the discrete notation for the continuous case. In particular, we will use italic bold symbols to indicate the dependence on ζ and will denote operators that act on the ζ dependence with non-italicized capital bold symbols. With this understanding,

29 $$\dot{p}(t)=-Kp(t)$$

will apply to both the continuous and discrete cases. Similarly, $1^{T}f$ will imply integration over the ζ dependence of the function f(ζ) in the continuous case and summation over all different states in the discrete case.

Note that in the probabilistic description of the random process by the Fokker–Planck equation (Eq. [Disp-formula Ch1.E28]), the probability density p(ζ,t) characterizes an ensemble of nuclei, and ζ is treated as an independent variable which is not a function of t . In contrast, when a single nucleus is followed in time (e.g., through molecular dynamics simulations), ζ is a random function of t . Although this second picture of random trajectories was invoked when writing the dipolar correlation function in Eq. ([Disp-formula Ch1.E2]), in the following pages we only work with the probabilistic description of an ensemble of identical nuclei.

Below, we will use the dynamical rule (Eq. [Disp-formula Ch1.E29]) when combining the stochastic dynamics of ζ with the spin dynamics from Sect. [Sec Ch1.S2]. The combined dynamics will be described by a stochastic Liouville equation (SLE) for a ζ -conditioned spin variable. In the case of the nuclear polarization, for example, the SLE will describe the dynamics of $P_{I}(t)$ , which stands for $P_{I}(\zeta ,t)$ in the continuous case. For a detailed explanation of SLE the reader is referred to the literature [Bibr bib1.bibx25]. A more recent discussion can be found in [Bibr bib1.bibx26].

### 3.2 Rate equations in liquids

#### 3.2.1 Electronic polarization

The electronic polarization was assumed to be insensitive to the dipolar coupling with the nuclear spins. Hence, the ζ -conditioned electronic polarization $P_{S}(t)$ is of the following separable form:

30 $$P_{S}(t)=p^{\mathrm{eq}}P_{S}(t),$$

in which all ζ dependence is isolated in the equilibrium probability of the stochastic process. From $P_{S}(t)$ we obtain the averaged (over ζ ) electronic polarization by summing/integrating over the ζ dependence. This is done with the help of the constant vector/function 1 as follows:

31 $$1^{T}P_{S}(t)=1^{T}p^{\mathrm{eq}}P_{S}(t)=P_{S}(t).$$

(In the last equality we used the normalization of the probability, $1^{T}p^{\mathrm{eq}}=1$ , which reads $\int d\zeta \;p^{\mathrm{eq}}(\zeta )=1$ in the continuous case.) Note that $P_{S}(t)$ in Eq. ([Disp-formula Ch1.E30]) is, in fact, the electronic polarization averaged over the stochastic variable (Eq. [Disp-formula Ch1.E31]).

In this description, the experimentally accessible polarizations correspond to the averaged values, while the ζ -dependent variables, like $P_{S}(t)$ , serve only an intermediate, book-keeping role. In other words, at the end we will always average over ζ by using the constant vector/function 1 .

Since Eq. ([Disp-formula Ch1.E30]) holds in general for the electronic polarization, it also holds at steady state and at equilibrium:

32 $$P_{S}^{\mathrm{ss}}=p^{\mathrm{eq}}P_{S}^{\mathrm{ss}},\;P_{S}^{\mathrm{eq}}=p^{\mathrm{eq}}P_{S}^{\mathrm{eq}}.$$

Here $P_{S}^{\mathrm{ss}}$ and $P_{S}^{\mathrm{eq}}$ are the averaged (over ζ ) values which correspond to the macroscopic polarization.

Lastly, we point out that at equilibrium all joint spin- ζ properties are of the above separable form. In other words, the last equality in Eq. ([Disp-formula Ch1.E32]) is not limited to the electronic polarization but applies to all other equilibrium properties.

#### 3.2.2 Nuclear polarization

To illustrate the SLE formalism and to introduce further notation, we start by transforming the rate equation of the nuclear polarization (Eq. [Disp-formula Ch1.E6]) to an SLE:

33 $$\begin{array}{rl} {\dot{P}}_{I}(t) & =-KP_{I}(t)-R_{1I}\left(P_{I}(t)-p^{\mathrm{eq}}P_{I}^{\mathrm{eq}}\right)\\ & \;-V_{+}P_{I}(t)-V_{-}p^{\mathrm{eq}}P_{S}(t). \end{array}$$

There are several different things going on here, so let us examine them one by one.

First, following the convention introduced above, $P_{I}(t)$ stands for $P_{I}(\zeta ,t)$ , which is the nuclear polarization conditional on the random state ζ . In this case the dot indicates partial derivative with respect to the time dependence, at fixed ζ . Second, the term $KP_{I}$ drives the dynamics in the ζ space by providing “off-diagonal” elements that mix the different random states. All remaining terms on the right-hand side of the SLE are “diagonal” in the ζ space and act only on the spin degree(s) of freedom (which are conditioned on ζ ). Third, the mw excitation rates $v_{\pm }$ and the relaxation rate $R_{1I}$ have acquired ζ dependence, turning into operators in ζ space that act on $P_{I}(t)$ or $p^{\mathrm{eq}}$ . In the discrete case, these would be matrices with different $v_{\pm }$ and $R_{1I}$ values for each discrete state ζ along their main diagonals. We use hollow capital letters to denote such “diagonal” operators in ζ space, also in the continuous case. Fourth, as all equilibrium properties, the nuclear Boltzmann polarization is separable, with the ζ dependence confined to the equilibrium probability of the random process.

The steady state of Eq. ([Disp-formula Ch1.E33]) is

34 $$\left(K+R_{1I}+V_{+}\right)P_{I}^{\mathrm{ss}}=\left(P_{I}^{\mathrm{eq}}R_{1I}-P_{S}^{\mathrm{eq}}pV_{-}\right)p^{\mathrm{eq}},$$

where we used $P_{S}^{\mathrm{ss}}=pP_{S}^{\mathrm{eq}}$ (Eq. [Disp-formula Ch1.E5]). Our aim is to solve Eq. ([Disp-formula Ch1.E34]) for $P_{I}^{\mathrm{ss}}$ and then obtain the macroscopic nuclear polarization by calculating the average $P_{I}^{\mathrm{ss}}=1^{T}P_{I}^{\mathrm{ss}}$ .

Clearly, solving Eq. ([Disp-formula Ch1.E34]) consists of calculating the inverse of the operator $\left(K+R_{1I}+V_{+}\right)$ . This is a daunting task in general and requires the matrix representation of K in some basis set. Here we will limit the discussion to random motions that are orders of magnitude faster than the nuclear $T_{1}$ relaxation rate, which we concluded to be the case even in viscous liquids like glycerol. This assumption ensures that, at steady state, all nuclear spins in the sample are equivalent and have the same polarization. Hence, we will look for a separable steady-state solution of the form

35 $$P_{I}^{\mathrm{ss}}=p^{\mathrm{eq}}P_{I}^{\mathrm{ss}}\;(\text{ansatz for liquids}).$$

With this ansatz, Eq. ([Disp-formula Ch1.E34]) becomes

36 $$\left(K+R_{1I}+V_{+}\right)p^{\mathrm{eq}}P_{I}^{\mathrm{ss}}=\left(P_{I}^{\mathrm{eq}}R_{1I}-P_{S}^{\mathrm{eq}}pV_{-}\right)p^{\mathrm{eq}}.$$

While the difference between Eqs. ([Disp-formula Ch1.E34]) and ([Disp-formula Ch1.E36]) appears to be minor, in fact we have achieved a tremendous simplification since $Kp^{\mathrm{eq}}=0$ , and thus the dynamical aspect of the random process is gone; only its equilibrium (i.e., time-independent) properties remain. Indeed, since in Eq. ([Disp-formula Ch1.E36]) all ζ operators act on the equilibrium probability $p^{\mathrm{eq}}$ , integration over the ζ dependence brings the average values:

37 $$\langle R_{1I}\rangle =1^{T}R_{1I}p^{\mathrm{eq}},\;\langle V_{\pm }\rangle =1^{T}V_{\pm }p^{\mathrm{eq}}.$$

(These are the average values of the ζ -dependent functions $R_{1I}(\zeta )$ and $v_{\pm }(\zeta )$ , respectively.) After averaging, Eq. ([Disp-formula Ch1.E36]) becomes

38 $$\left(\langle R_{1I}\rangle +\langle V_{+}\rangle \right)P_{I}^{\mathrm{ss}}=\langle R_{1I}\rangle P_{I}^{\mathrm{eq}}-p\langle V_{-}\rangle P_{S}^{\mathrm{eq}}.$$

Comparison of Eqs. ([Disp-formula Ch1.E38]) and ([Disp-formula Ch1.E7]) shows that the phenomenological rates $R_{1I}$ and $v_{\pm }$ in the rate equation should be identified with the macroscopic averages $\langle R_{1I}\rangle$ and $\langle V_{\pm }\rangle$ over the liquid sample. This is the familiar regime of fast motion, where one observes the averaged values of the magnetic parameters. We have thus provided a formal justification of why the averaged $\delta^{2}$ in Eq. ([Disp-formula Ch1.E27]) corresponds to fast spin diffusion in the case of solids.

We observe that the static averages over 3D space, which are implied by Eq. ([Disp-formula Ch1.E37]), do not allow for the partial dynamical averaging of the dipolar interaction. As discussed above, such averaging should be based on the time-correlation function of $A_{1}$ . However the rate constants $v_{\pm }$ always contain the square of the dipolar interaction and do not provide access to $A_{1}$ itself. We thus conclude that the partial averaging of the dipolar interaction in liquids is inaccessible to modeling by rate equations.

### 3.3 Spin dynamics in liquids

To gain access to the dipolar interaction before it is squared, we turn to the equations of motion of the coherences from Sect. [Sec Ch1.S2.SS3]. We first transform the equation of $i_{z}$ (Eq. [Disp-formula Ch1.E14]) to an SLE:

39 i˙z(t)|coh=-Kiz(t)-Re{iA1*gz(t)}.

As before, $i_{z}(t)$ and $g_{z}(t)$ stand for $i_{z}(\zeta ,t)$ and $g_{z}(\zeta ,t)$ in the continuous case, K acts on the ζ dependence of $i_{z}$ and $A_{1}^{*}$ has become a “diagonal” operator in ζ space. Averaging Eq. ([Disp-formula Ch1.E39]) over ζ , we obtain the macroscopic equation

40 1Ti˙z(t)|coh=-Re{i1TA1*gz(t)}.

At steady state, using the ansatz for liquids (Eq. [Disp-formula Ch1.E35]) in the form $i_{z}^{\mathrm{ss}}=p^{\mathrm{eq}}i_{z}^{\mathrm{ss}}$ , we have

41 i˙z|cohss=-Re{i1TA1*gzss}.

Since we accounted only for the coherent contribution to the time derivative of $i_{z}$ , the right-hand side of Eq. ([Disp-formula Ch1.E41]) corresponds to the right-hand side of Eq. ([Disp-formula Ch1.E7]). Our aim is to identify the phenomenological rate constants $v_{\pm }$ that should be used in Eq. ([Disp-formula Ch1.E7]) by analyzing Re{i1TA1*gzss} . However, because the random modulation of $A_{1}$ additionally contributes to the nuclear $T_{1}$ relaxation, we have the equality

42 Re{i1TA1*gzss}=R1IAizss+v+izss+pv-szeq

from which we will read out the desired rates.

#### 3.3.1 Contribution to nuclear $T_{1}$ relaxation

The contribution of Re{i1TA1*gzss} to the nuclear spin-lattice relaxation can be identified by its value in the absence of mw irradiation. From Eqs. ([Disp-formula Ch1.E12]) and ([Disp-formula Ch1.E13]) we see that for $\omega_{1}=0$ the dynamics of $g_{z}$ completely decouples from $g_{x}$ and $g_{y}$ . The SLE of $g_{z}$ in this case becomes

43 $${\dot{g}}_{z}(t)=-Kg_{z}(t)-(R_{1S}+i\omega_{I})g_{z}(t)-i\frac{1}{4}A_{1}i_{z}(t).$$

Technically, $R_{1S}$ and $\omega_{I}$ should be operators that act on the ζ dependence of $g_{z}$ . However, we take the electronic $T_{1}$ relaxation rate and the nuclear Larmor frequency to be independent of the dipolar coupling, which is parametrized by ζ . The corresponding operators are then $R_{1S}I$ and $\omega_{I}I$ , where I is the identity operator in ζ space. This identity operator will not be written explicitly.

The steady-state solution of Eq. ([Disp-formula Ch1.E43]) is

44 $$g_{z}^{\mathrm{ss}}=-i\frac{1}{4}(K+R_{1S}+i\omega_{I}{)}^{-1}A_{1}p^{\mathrm{eq}}i_{z}^{\mathrm{ss}},$$

where we again used the ansatz for liquids. Substituting this $g_{z}^{\mathrm{ss}}$ on the left-hand side of Eq. ([Disp-formula Ch1.E42]), we find that the nuclear relaxation rate due to $A_{1}$ is

45 R1IA=14Re1TA1*K+R1S+iωI-1A1peq.

To express this relaxation rate in a more intelligible manner, we observe that the inverse of a matrix M whose eigenvalues have strictly positive real parts can be written as

46 $$M^{-1}=\int_{0}^{\infty }e^{-Mt}\;dt.$$

Applying this identity to the matrix $\left(K+R_{1S}+i\omega_{I}\right)$ in Eq. ([Disp-formula Ch1.E45]), we find

47 R1IA=14Re∫0∞dte-(R1S+iωI)tC11(t),

where

48 $$\begin{array}{rl} C_{11}(t) & =1^{T}A_{1}^{*}e^{-Kt}A_{1}p^{\mathrm{eq}}\\ & =\int d\zeta \;A_{1}^{*}(\zeta )e^{-K_{\zeta }t}A_{1}(\zeta )p^{\mathrm{eq}}(\zeta ) \end{array}$$

is the time-correlation function of the dipolar interaction (Eq. [Disp-formula Ch1.E2]). Since the integral in Eq. ([Disp-formula Ch1.E47]) corresponds to the Laplace transform

49 $$J_{11}(s)=\int_{0}^{\infty }dt\;e^{-st}C_{11}(t),$$

we have

50 R1IA=14ReJ11(R1S+iωI).

The real part of the Laplace transform is known as spectral density. Here the spectral density is evaluated at a the complex argument $R_{1S}+i\omega_{I}$ , which contains both the nuclear Larmor frequency and the electronic $T_{1}$ relaxation rate.

Let us examine Eq. ([Disp-formula Ch1.E50]) in the solid-state limit where $A_{1}$ does not change with time. Then $C_{11}=\langle A_{1}^{*}A_{1}\rangle$ and

51 $$R_{1I,\mathrm{solid}}^{A}=\langle \delta^{2}\rangle \frac{T_{1S}}{1+T_{1S}^{2}\omega_{I}^{2}}.$$

In Abragam's nomenclature [Bibr bib1.bibx1] this is relaxation of the second kind, meaning that it is due to the relaxation of the electronic spins and not due to the modulation of the dipolar interaction by motion. This $R_{1I,\mathrm{solid}}^{A}/\langle \delta^{2}\rangle$ was shown with horizontal, dotted red lines in the third row of Fig. [Fig Ch1.F3].

In the case of liquids, we expect $C_{11}(t)$ to decay with time. Assuming a mono-exponential decay with correlation time τ ,

52 R1I,expA=〈δ2〉Re∫0∞dte-R1S+iωI+τ-1t=〈δ2〉ReR1S+τ-1+iωI-1.

This $R_{1I,\exp}^{A}/\langle \delta^{2}\rangle$ was shown with horizontal, dotted black lines in the third row of Fig. [Fig Ch1.F3]. To understand why it increases with decreasing $\omega_{I}$ , let us examine the case of motion that is faster than the electronic $T_{1}$ time, i.e., $\tau^{-1}\gg R_{1S}$ . The result,

53 $$R_{1I,\exp}^{A}\approx \langle \delta^{2}\rangle \frac{\tau }{1+\tau^{2}\omega_{I}^{2}},$$

is relaxation of the first kind with Lorentzian spectral density. Clearly, smaller $\omega_{I}$ implies larger dipolar contribution to the nuclear $T_{1}$ relaxation rate.

Having identified the relaxation rate $R_{1I}^{A}$ on the right-hand side of Eq. ([Disp-formula Ch1.E42]), we now proceed with the analysis of the rates $v_{\pm }$ characterizing the forbidden transitions.

#### 3.3.2 Contribution to forbidden transitions

Combining the dynamics of the coherences (Eq. [Disp-formula Ch1.E12]) with the stochastic dynamics (Eq. [Disp-formula Ch1.E29]), we arrive at the following SLE:

54 $$\left[\begin{array}{c} {\dot{g}}_{x}\\ {\dot{g}}_{y}\\ {\dot{g}}_{z} \end{array}\right]=-\left(K+B\right)\left[\begin{array}{c} g_{x}\\ g_{y}\\ g_{z} \end{array}\right]-i\frac{1}{4}A_{1}\left[\begin{array}{c} -is_{y}p^{\mathrm{eq}}\\ is_{x}p^{\mathrm{eq}}\\ i_{z} \end{array}\right].$$

Note that the matrix B does not depend on time as the electronic relaxation properties were taken to be insensitive to the dipolar interaction between the electronic and nuclear spins. Recall that the operators written as upright bold letters (including the hollow ones) act on the ζ dependence of the variables, which is encoded by the italic bold symbols. The script uppercase letters denote 3×3 matrices, which act on the column vectors that are shown explicitly.

Although not shown explicitly in Eq. ([Disp-formula Ch1.E54]), we imply the tensor products of the operators K , B and $A_{1}$ with the identity operators in the spaces on which K , B and $A_{1}$ do not act (i.e., K and $A_{1}$ are in fact I⊗K and $I⊗A_{1}$ where I is the 3×3 identity matrix, and B is B⊗I where I is the identity operator in the ζ space).

Before solving Eq. ([Disp-formula Ch1.E54]) at steady state, let us introduce the (right) eigenvalue problem of B ,

55 BU=UD,

where the diagonal matrix

56 $$D=\text{diag}\left(\lambda_{1},\lambda_{2},\lambda_{3}\right)$$

contains the eigenvalues of B along its main diagonal, and the columns of the 3×3 matrix U contain the corresponding right eigenvectors. Then, the steady state of Eq. ([Disp-formula Ch1.E54]) is

57 $$U\left(K+D\right)U^{-1}\left[\begin{array}{c} g_{x}^{\mathrm{ss}}\\ g_{y}^{\mathrm{ss}}\\ g_{z}^{\mathrm{ss}} \end{array}\right]=-i\frac{1}{4}A_{1}p^{\mathrm{eq}}\left[\begin{array}{c} -is_{y}^{\mathrm{ss}}\\ is_{x}^{\mathrm{ss}}\\ i_{z}^{\mathrm{ss}} \end{array}\right],$$

which, after inverting the matrices, yields

58 $$\left[\begin{array}{c} g_{x}^{\mathrm{ss}}\\ g_{y}^{\mathrm{ss}}\\ g_{z}^{\mathrm{ss}} \end{array}\right]=-i\frac{1}{4}U{\left(K+D\right)}^{-1}A_{1}p^{\mathrm{eq}}U^{-1}\left[\begin{array}{c} -is_{y}^{\mathrm{ss}}\\ is_{x}^{\mathrm{ss}}\\ i_{z}^{\mathrm{ss}} \end{array}\right].$$

Plugging this solution for $g_{z}^{\mathrm{ss}}$ into the left-hand side of Eq. ([Disp-formula Ch1.E42]), and defining the matrix

59 $$L=1^{T}A_{1}^{*}{\left(K+D\right)}^{-1}A_{1}p^{\mathrm{eq}},$$

we find

60 Re{i1TA1*gzss}=14Re{001ULU-1-isyssisxssizss}.

Comparison with the right-hand side of Eq. ([Disp-formula Ch1.E42]) yields

61 R1IA+v+=14001ReULU-1001Tv-=14001ReiULU-1ω1fyω1fx0T,

where we used the relationships between $s_{x,y}^{\mathrm{ss}}$ and $s_{z}^{\mathrm{eq}}$ (Eq. [Disp-formula Ch1.E15]) to arrive at $v_{-}$ .

We observe that L is a 3×3 diagonal matrix without any ζ dependence since the right-hand side of Eq. ([Disp-formula Ch1.E59]) is averaged over ζ . With $L=\text{diag}(L_{1},L_{2},L_{3})$ , we have

62 $$L_{n}=1^{T}A_{1}^{*}{\left(K+\lambda_{n}\right)}^{-1}A_{1}p^{\mathrm{eq}}\;(n=1,2,3).$$

Using Eq. ([Disp-formula Ch1.E46]), these diagonal elements can be written as

63 $$\begin{array}{rl} L_{n} & =1^{T}A_{1}^{*}\int_{0}^{\infty }e^{-\left(K+\lambda_{n}\right)t}\;dt\;A_{1}p^{\mathrm{eq}}\\ & =\int_{0}^{\infty }e^{-\lambda_{n}t}\;\left(1^{T}A_{1}^{*}e^{-Kt}A_{1}p^{\mathrm{eq}}\right)\;dt\\ & =\int_{0}^{\infty }e^{-\lambda_{n}t}\;C_{11}(t)\;dt=J_{11}\left(\lambda_{n}\right), \end{array}$$

where we used Eq. ([Disp-formula Ch1.E48]) in the third equality and Eq. ([Disp-formula Ch1.E49]) in the last one. Hence, each $L_{n}$ is the Laplace transform of the time-correlation function $C_{11}(t)$ evaluated at the eigenvalue $\lambda_{n}$ of B . The matrix L to be used in Eq. ([Disp-formula Ch1.E61]) is thus

64 $$L=\text{diag}\left(J_{11}\left(\lambda_{1}\right),J_{11}\left(\lambda_{2}\right),J_{11}\left(\lambda_{3}\right)\right).$$

In summary, for any given set of parameters, we form the 3×3 matrix B (Eq. [Disp-formula Ch1.E13]) and numerically calculate its eigenvalues and right eigenvectors. The former are used in Eq. ([Disp-formula Ch1.E64]) to calculate L . Sandwiching L by the eigenvectors, as required in Eq. ([Disp-formula Ch1.E61]), we arrive at the desired rates $v_{\pm }$ . This prescription applies to any motional model describing the stochastic dynamics of the inter-spin vector. Different models will differ only in their spectral densities $J_{11}$ .

From a mathematical point of view, the simplest case is a model with exponential dipolar correlation function, $C_{11}^{\exp}(t)=\langle \delta^{2}\rangle e^{-t/\tau }$ , where τ is the correlation time. Then

65 $$J_{11}^{\exp}(s)=\langle \delta^{2}\rangle \;\frac{1}{s+\frac{1}{\tau }}=\langle \delta^{2}\rangle \;\tau \frac{1}{s\tau +1},$$

and Eq. ([Disp-formula Ch1.E64]) becomes

66 $$L_{\exp}=\langle \delta^{2}\rangle \;\text{diag}\left(\frac{1}{\lambda_{1}+\frac{1}{\tau }},\frac{1}{\lambda_{2}+\frac{1}{\tau }},\frac{1}{\lambda_{3}+\frac{1}{\tau }}\right).$$

All dashed black lines labeled “liquid” in Fig. [Fig Ch1.F3] were calculated using Eq. ([Disp-formula Ch1.E66]) with τ=12 ns.

Comparing $v_{+}$ and $pv_{-}$ (Fig. [Fig Ch1.F3], third and fourth rows) between the solid and liquid cases, we see that at Q, W and J bands, the fluctuations of the dipolar interaction have substantially broadened the lines centered at the canonical solid-effect offsets $\Omega \approx \pm \omega_{I}$ and have reduced the peak enhancements in liquids compared to solids (fifth row). At X band, where the two lines had already merged in the solid case, the effect of fluctuations is qualitatively different, although line broadening is also visible. Most strikingly, the rate $v_{+}$ is seen to become negative at offsets larger than $\omega_{I}$ , which leads to a nuclear polarization factor (Eq. [Disp-formula Ch1.E9]) that exceeds 1 (Fig. [Fig Ch1.F3], bottom row).

## 4 Closer look at liquids

### 4.1 Translational diffusion of hard spheres

A mono-exponential dipolar correlation function is a poor model of translational diffusion in liquids. The so-called force-free hard-sphere (FFHS) model, which assumes spherical molecules that contain the spins at their centers, is a more realistic yet analytically tractable model [Bibr bib1.bibx4]. It is universally employed in the analysis of diverse magnetic-resonance measurements, including nuclear relaxation by paramagnetic impurities [Bibr bib1.bibx33] and DNP via the Overhauser effect [Bibr bib1.bibx11].

Because the spins are taken to be at the centers of the spherical molecules, the FFHS model has only two parameters: the coefficient of translational diffusion, D , and the distance of the spins upon contact of the spherical molecules, b . These two parameters form the characteristic motional timescale of the model [Bibr bib1.bibx4]:

67 $$\tau =b^{2}/D.$$

The Laplace transform of the dipolar correlation function of this model is Eqs. 51 and 55

68 $$J_{11}^{\mathrm{ffhs}}(s)=\langle \delta^{2}\rangle \;\tau \;\frac{(s\tau {)}^{\frac{1}{2}}+4}{(s\tau {)}^{\frac{3}{2}}+4(s\tau )+9(s\tau {)}^{\frac{1}{2}}+9}.$$

Using $J_{11}^{\mathrm{ffhs}}$ in Eq. ([Disp-formula Ch1.E64]) we calculated numerically the same properties as in Fig. [Fig Ch1.F3] but for the FFHS model. The results are shown with colored solid lines in Fig. [Fig Ch1.F4]. For comparison, the model with mono-exponential correlation function from Fig. [Fig Ch1.F3] is also reproduced in Fig. [Fig Ch1.F4] with dashed black lines.

**Figure 4 Ch1.F4:**
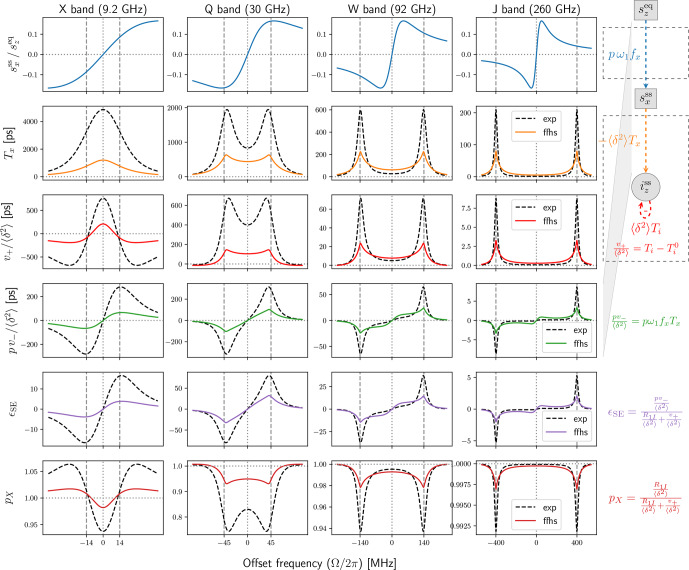
Same as Fig. [Fig Ch1.F3] for the model with exponential time-correlation function (dashed black lines) and the FFHS model (colored solid lines) both with $\tau =T_{2S}/5=12$ ns.

The general observation from Fig. [Fig Ch1.F3] that the fluctuation of the dipolar interaction broadens the solid-effect lines at $\Omega \approx \pm \omega_{I}$ is even more relevant for the FFHS model. Indeed, for the same dipolar timescale τ , the FFHS lines are much broader and, correspondingly, much smaller in peak amplitude than the lines of the exponential model. Hence, the FFHS model predicts significantly smaller DNP enhancements (Fig. [Fig Ch1.F4], second last row) compared to the exponential model with the same timescale τ . At X band, the negative values of $v_{+}$ are still present, but their magnitude is substantially reduced (third row). The corresponding offsets where the nuclear polarization factor, $p_{X}$ , is larger than 1 are similar in the two models, but again the deviation from 1 is much smaller in the FFHS model (last row).

Overall, $p_{X}$ in liquids is very close to 1 (last row of Fig. [Fig Ch1.F4], FFHS model), which indicates that $v_{+}$ is very small compared to $R_{1I}$ . In such cases, the solid-effect DNP enhancement (Eq. [Disp-formula Ch1.E9]) is well approximated by Eq. ([Disp-formula Ch1.E10]). This explains why the enhancement in the fifth row of Fig. [Fig Ch1.F4] is essentially a rescaled version of the row directly above it.

The substantial reduction of the peak intensities at the solid-effect offsets $\Omega \approx \pm \omega_{I}$ is accompanied by a smaller but still appreciable *increase* of the intensities at small offsets ( Ω≈0 ). This trend is visible both in the transition from the solid case to a mono-exponential correlation function (Fig. [Fig Ch1.F3], second row) and in the further transition to the FFHS model (Fig. [Fig Ch1.F4]). The significance of this observation will become clear in Sect. [Sec Ch1.S4.SS4], where we compare our calculations with the experiments of [Bibr bib1.bibx27].

### 4.2 Approximate matrix inversion

Since B is a 3×3 matrix, its eigenvalues and eigenvectors are easily determined numerically, as we did when calculating the exponential and FFHS models in Fig. [Fig Ch1.F4]. Nevertheless, to gain insight into the eigenvalue problem that is being solved, here we analyze Eq. ([Disp-formula Ch1.E55]) using perturbation theory. The analysis reveals that the eigenvalue problem is related to the effective magnetic field and the associated “tilted” coordinate frame [Bibr bib1.bibx42].

Let us introduce the matrix

69 $$B_{0}=\left[\begin{array}{ccc} R_{2S}+i\omega_{I} & \Omega & 0\\ -\Omega & R_{2S}+i\omega_{I} & \omega_{1}\\ 0 & -\omega_{1} & R_{2S}+i\omega_{I} \end{array}\right],$$

where $R_{1S}$ in the lower right corner of B (Eq. [Disp-formula Ch1.E13]) has been replaced by $R_{2S}$ . The three eigenvalues of $B_{0}$ are

70 $$\lambda_{0,0}=R_{2S}+i\omega_{I},\;\lambda_{0,\mp }=R_{2S}+i\left(\omega_{I}\mp \omega_{\mathrm{eff}}\right),$$

where the frequency

71 $$\omega_{\mathrm{eff}}=\sqrt{\Omega^{2}+\omega_{1}^{2}}$$

corresponds to the effective magnetic field in the rotating frame. This field is tilted away from the z axis by an angle α such that

72 $$\mathrm{cos}\alpha =\Omega /\omega_{\mathrm{eff}}=c,\;\mathrm{sin}\alpha =\omega_{1}/\omega_{\mathrm{eff}}=s.$$

With the sine and cosine of α abbreviated as s and c, the right eigenvectors of $B_{0}$ are

73 $$U_{0}=\left[\begin{array}{ccc} s & -c/\sqrt{2} & -c/\sqrt{2}\\ 0 & i/\sqrt{2} & -i/\sqrt{2}\\ c & s/\sqrt{2} & s/\sqrt{2} \end{array}\right],$$

where the first column corresponds to $\lambda_{0,0}$ , the second to $\lambda_{0,-}$ , and the third to $\lambda_{0,+}$ . By inspection, $U_{0}^{-1}=U_{0}^{H}$ , where the superscript “H” denotes Hermitian conjugation.

We treat the difference $B-B_{0}$ as a perturbation to $B_{0}$ . To first order in the perturbation, the eigenvalues of the original matrix B are ${\tilde{\lambda }}_{n}=\lambda_{0,n}+u_{0,n}^{H}(B-B_{0})u_{0,n}$ , where $u_{0,n}$ is the n th column of $U_{0}$ . Using this expression we find the corrected eigenvalues

74 $${\tilde{\lambda }}_{0}={\tilde{R}}_{1}+i\omega_{I},\;{\tilde{\lambda }}_{\mp }={\tilde{R}}_{2}+i\left(\omega_{I}\mp \omega_{\mathrm{eff}}\right),$$

with

75 $$\begin{array}{rl} {\tilde{R}}_{1} & =R_{1S}(\mathrm{cos}\alpha {)}^{2}+R_{2S}(\mathrm{sin}\alpha {)}^{2}\\ {\tilde{R}}_{2} & =R_{2S}[1-(\mathrm{sin}\alpha {)}^{2}/2]+R_{1S}(\mathrm{sin}\alpha {)}^{2}/2. \end{array}$$

Collecting the eigenvalues (Eq. [Disp-formula Ch1.E74]) in the diagonal matrix $\tilde{D}=\text{diag}({\tilde{\lambda }}_{0},{\tilde{\lambda }}_{-},{\tilde{\lambda }}_{+})$ , we have $B^{-1}\approx U_{0}{\tilde{D}}^{-1}U_{0}^{H}$ . As an example, the element in the lower right corner of the inverse matrix is

76 $$B_{33}^{-1}=F_{z}\approx (\mathrm{cos}\alpha {)}^{2}{\tilde{\lambda }}_{0}^{-1}+\frac{1}{2}(\mathrm{sin}\alpha {)}^{2}\left({\tilde{\lambda }}_{-}^{-1}+{\tilde{\lambda }}_{+}^{-1}\right).$$

(This approximation of $F_{z}$ was used in [Bibr bib1.bibx37] without proof.)

From Eq. ([Disp-formula Ch1.E76]), and using the first equality in Eq. ([Disp-formula Ch1.E22]), we immediately find

77 Ti≈Rec2λ~0-1+s2λ~--1+λ~+-1/2.

To obtain $v_{+}$ , we need to subtract $T_{i}^{0}$ from $T_{i}$ (Eq. [Disp-formula Ch1.E24]). Since $\omega_{1}=0$ implies s=0 and c=1 ,

78 Ti0=ReR1S+iωI-1,

which is identical to the exact result in Eq. ([Disp-formula Ch1.E25]). Hence,

79 v+/δ2≈Res2λ~--1+λ~+-1/2+c2λ~0-1-R1S+iωI-1.

One can similarly obtain the rate constant $v_{-}$ as a linear combination of the reciprocals of the approximate eigenvalues ${\tilde{\lambda }}_{0}$ and ${\tilde{\lambda }}_{\mp }$ . The result is

80 Tx=ω1ωeff2Re{iX},

with

81 $$X=R_{2S}{\tilde{\lambda }}_{0}^{-1}-\frac{1}{2}\left(R_{2S}+i\omega_{\mathrm{eff}}\right){\tilde{\lambda }}_{-}^{-1}-\frac{1}{2}\left(R_{2S}-i\omega_{\mathrm{eff}}\right){\tilde{\lambda }}_{+}^{-1}.$$

Recall that $v_{-}/\delta^{2}=(\omega_{1}f_{x})T_{x}$ (Eq. [Disp-formula Ch1.E24]).

The first eigenvalue in Eq. ([Disp-formula Ch1.E74]) does not depend on the offset Ω . The other two eigenvalues depend on the offset through $\omega_{\mathrm{eff}}$ . Let us consider sufficiently large offsets such that $|\Omega |\gg \omega_{1}$ , and so $\omega_{\mathrm{eff}}\approx |\Omega |$ . This condition is satisfied at the solid-effect offset positions $\Omega \approx \pm \omega_{I}$ at W and J bands but may be entirely inapplicable to X band at large mw powers, as discussed in [Bibr bib1.bibx37]. When the condition holds, s≈0 and c≈1 , and the eigenvalues in Eq. ([Disp-formula Ch1.E74]) become ${\tilde{\lambda }}_{0}\approx R_{1S}+i\omega_{I}$ and ${\tilde{\lambda }}_{\mp }\approx R_{2S}+i(\omega_{I}\mp |\Omega |)$ . Thus ${\tilde{\lambda }}_{\mp }^{-1}$ correspond to complex-valued Lorentzians centered at $\Omega =\pm \omega_{I}$ and with widths equal to the homogeneous EPR line width (without power broadening). These are the Lorentzians that we see as narrow lines at W and J bands in the second and third rows of Fig. [Fig Ch1.F3] (orange and red solid lines).

In the case of motion, assuming mono-exponential correlation function for simplicity, each eigenvalue is replaced by ${\tilde{\lambda }}_{n}+1/\tau$ . This amounts to increasing the widths of the solid-effect Lorentzians from $R_{2S}$ to $R_{2S}+1/\tau$ . The resulting motional broadening is the reason for the differences between the “solid” and “liquid” lines in the second and third rows of Fig. [Fig Ch1.F3].

**Figure 5 Ch1.F5:**
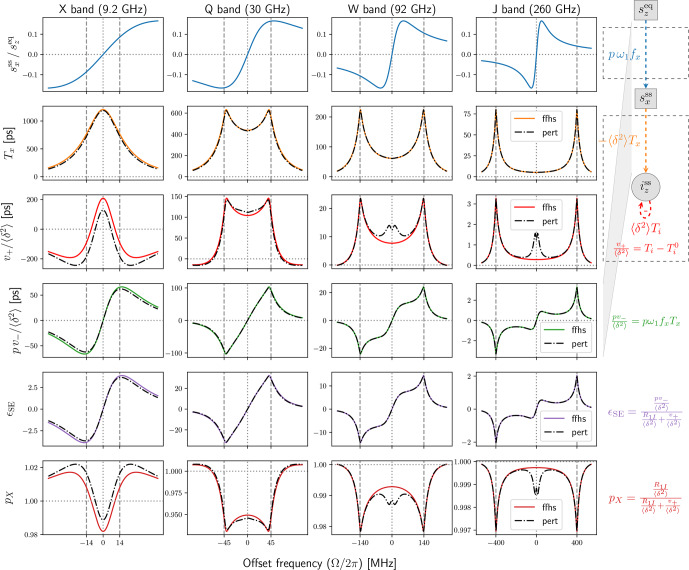
Comparison between the exact (solid colored lines) and perturbative (dashed black lines) calculations of the FFHS model. All parameters as in Fig. [Fig Ch1.F4]. The eigenvalue problem of the generalized Bloch matrix B has a simple closed-form solution when $T_{1S}=T_{2S}$ . The perturbative approximation uses these analytical eigenvectors and corrects the eigenvalues to first order in the difference $1/T_{1S}-1/T_{2S}$ .

For a general motional model, we have the approximate $\tilde{L}=\text{diag}(J_{11}({\tilde{\lambda }}_{0}),J_{11}({\tilde{\lambda }}_{-}),J_{11}({\tilde{\lambda }}_{+}))$ , which yields the approximation $ULU^{-1}\approx U_{0}\tilde{L}U_{0}^{H}$ to be used in Eq. ([Disp-formula Ch1.E61]). For the spectral density of the FFHS model, the perturbative expressions are compared with the exact numerical calculation in Fig. [Fig Ch1.F5]. The former are plotted with dashed–dotted black lines and the latter with colored solid lines, like in Fig. [Fig Ch1.F4]. We see that the perturbative analysis is satisfactory in general, at least for the specific choice of parameters that were used. It gives excellent predictions for $v_{-}$ (Fig. [Fig Ch1.F5], fourth row) and, because the two are related by a global scaling factor (Eq. [Disp-formula Ch1.E10]), also for the DNP enhancement (fifth row). At the same time, it is seen to consistently fail for the rate $v_{+}$ at small offsets in the vicinity of the origin (third row).

We should mention that the perturbative approximation becomes progressively better when $R_{2S}$ approaches $R_{1S}$ (not shown), as it is exact for $R_{2S}=R_{1S}$ .

Leaving the approximation quality of the perturbative analysis aside, we observe that the enhancement profiles in the fifth row of Fig. [Fig Ch1.F5] reveal the emergence of a novel feature at small offsets. At W band, this feature appears as a shoulder in the broadened lines, and at J band it is already separated from the canonical solid-effect peaks. Comparison with the lines in the first row of Fig. [Fig Ch1.F5] makes it clear that this new feature in the DNP spectrum coincides with the extrema of the dispersive component of the power-broadened EPR line. For saturating mw powers, where $\omega_{1}\gg R_{1S}R_{2S}$ , these extrema are at

82 $$\Omega_{1/2}=\omega_{1}\sqrt{T_{1S}/T_{2S}}.$$

(The subscript 1/2 was selected because these are also the offset positions where the electronic saturation factor equals one half.) The factor $pv_{-}$ in the fourth row of Fig. [Fig Ch1.F5] is obtained as the product of the first and second rows, as elaborated in [Bibr bib1.bibx37]. When the solid-effect lines at $\Omega \approx \pm \omega_{I}$ (second row) become sufficiently broad, their amplitude at $\Omega_{1/2}$ gets large enough for the peak of the dispersive EPR line (first row) to be visible in the DNP spectrum.

### 4.3 Motional suppression and broadening

Let us examine more closely the suppression of the lines at the solid-effect offsets and the concurrent increase of their intensity at $\Omega_{1/2}$ . We will limit the discussion to J band where the condition $\omega_{I}\gg \omega_{1}$ holds, and the solid-effect offsets are $\Omega =\pm \omega_{I}$ . Using the perturbative eigenvalues in Eq. ([Disp-formula Ch1.E74]), we see that only the real parts of ${\tilde{\lambda }}_{\mp }$ survive at these offsets. The peak amplitudes of the solid-effect lines are then proportional to $J_{11}(R_{2S})$ .

The limits $\tau \ll T_{2S}$ and $\tau \gg T_{2S}$ correspond to, respectively, very fast and very slow diffusive motion relative to the electronic $T_{2}$ . In the slow limit $\tau \gg T_{2S}$ , we have

83 $$\underset{R_{2S}\tau \to \infty }{lim}J_{11}\left(R_{2S}\right)/\langle \delta^{2}\rangle \to T_{2S}$$

for both the mono-exponential and FFHS motional models. This means that, in solids, the peaks increase with the electronic $T_{2}$ . In the opposite limit of very fast motion, i.e., $\tau \ll T_{2S}$ , we find

84 $$\underset{R_{2S}\tau \to 0}{lim}\frac{J_{11}\left(R_{2S}\right)}{\langle \delta^{2}\rangle }\to \left\{\begin{array}{cc} \tau & \mathrm{exponential}\\ \frac{4}{9}\tau & \mathrm{FFHS} \end{array}\right.$$

which means that, in liquids, the peak amplitudes are proportional to the dipolar correlation time τ . In other words, faster fluid diffusion (i.e., smaller τ ) corresponds to smaller peaks and thus smaller solid-effect enhancement at the canonical offsets. We also see that for the same τ the peaks of the FFHS model are less than half of the peaks of the exponential model, which is in agreement with Fig. [Fig Ch1.F4] (second and third rows).

To describe the transition between the fast and slow limits, we define the reduction factor

85 $$\rho (\tau )=\frac{1}{T_{2S}}\frac{J_{11}(R_{2S};\tau )}{\langle \delta^{2}\rangle }$$

which equals one in the solid limit and approaches zero for small τ . Since it quantifies how much smaller the peaks are compared to the solid case, ρ is a measure of how “solid-like” the liquid is.

**Figure 6 Ch1.F6:**
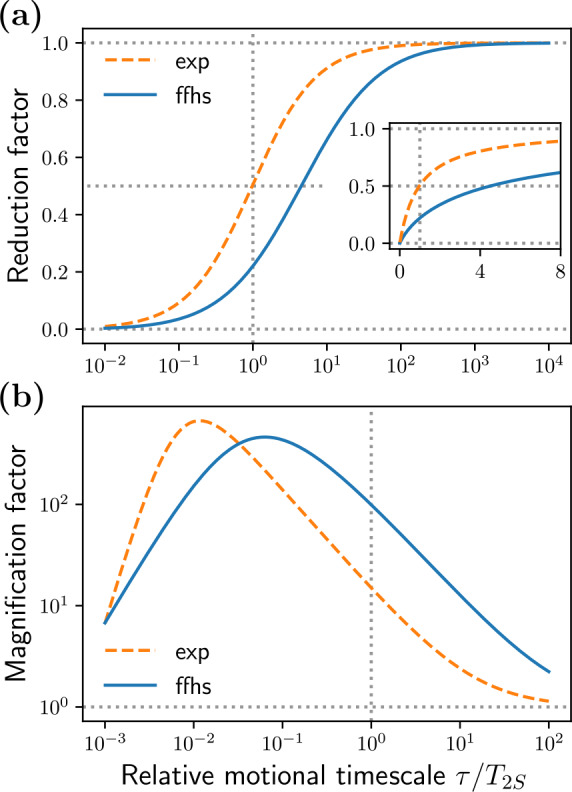
Multiplicative deviation of $T_{x}(\Omega )$ from the solid limit at **(a)** the solid-effect offsets $\Omega =\pm \omega_{I}$ and **(b)** the offsets $\Omega_{1/2}=\pm \omega_{1}\sqrt{T_{1S}/T_{2S}}$ for the exponential and FFHS models.

Figure [Fig Ch1.F6]a shows the reduction factors of the exponential and FFHS models against the relative motional timescale $\tau /T_{2S}$ . For the exponential model, the solid-effect peaks drop to half of their maximum values at $\tau =T_{2S}$ . In the case of the FFHS model, this happens already at $\tau \approx 4T_{2S}$ (see inset). In other words, appreciable reduction compared to the solid limit occurs even for exceedingly long diffusive timescales, several-fold compared to the electronic $T_{2}$ . For identical τ 's the exponential model is seen to be more solid-like than the diffusive FFHS model across the entire motional range. Hence, realistic translational diffusion suppresses the solid-effect peaks more effectively than mono-exponential decay.

As a quantitative measure of the motional broadening, let us consider the magnitude of $T_{x}$ (Fig. [Fig Ch1.F5], second row) at the locations of the extrema of the dispersive EPR line (first row). Since the intensity at these small offsets increases when going from the solid to the liquid case, we define the magnification factor

86 $$\mu (\tau )=\frac{T_{x}^{\mathrm{liquid}}(\Omega_{1/2};\tau )}{T_{x}^{\mathrm{solid}}(\Omega_{1/2})}.$$

This factor is shown in Fig. [Fig Ch1.F6]b. For the FFHS model, the intensity at $\Omega_{1/2}$ is 2 to 3 orders of magnitude larger than the solid case across a broad range of motional timescales, between $\tau =T_{2S}$ and $\tau =0.01T_{2S}$ . Hence the peak of the dispersive EPR line should be magnified 100- to 1000-fold in liquids compared to the solid limit. It is also magnified for the mono-exponential dipolar correlation function, although not to the same extent.

In the light of these observations, next we analyze the DNP field profile of recent experiments with the free radical BDPA in DMPC lipid bilayers at 320 K [Bibr bib1.bibx27].

### 4.4 Comparison with experiment

The DNP experiments of [Bibr bib1.bibx27] were carried out at J band (260 GHz/400 MHz). For the acyl chain protons of the DMPC lipids, the peak DNP enhancements at the canonical solid-effect offsets were ±12 [Bibr bib1.bibx27]. Two additional enhancement peaks of ±8 were also observed at much smaller offsets. These were attributed to thermal mixing. Here we argue that they correspond to the extrema of the dispersive component of the EPR line.

The enhancements in [Bibr bib1.bibx27] were for a BDPA-to-lipid ratio of 1:10 at a temperature of about 320 K. The room-temperature EPR spectrum of BDPA at J band for this relatively high radical concentration was very narrow Fig. 2. The transverse relaxation time implied by this narrow line is $T_{2S}=215$ ns. For the same radical concentration, the nuclear spin-lattice relaxation time at J band was 50 ms at 298 K [Bibr bib1.bibx27]. Although the experimental $T_{1I}$ and $T_{2S}$ are for 298 K, below we use these values to fit the DNP spectrum at 320 K. We also use $B_{1}=6$ G, as estimated in [Bibr bib1.bibx27].

In addition to these parameters with experimental support, three more parameters are needed for the calculation of the DNP enhancement: $T_{1S}$ , τ and $N/3b^{3}$ . We will treat these as fitting parameters. Let us introduce the ratios

87 $$r_{1}=\frac{T_{1S}}{T_{2S}},\;r_{2}=\frac{T_{2S}}{\tau },\;r_{3}=\frac{N/3b^{3}}{N_{\mathrm{ref}}/3b_{\mathrm{ref}}^{3}}.$$

The ratio $r_{1}$ expresses the unknown electronic $T_{1}$ relaxation time in terms of the known electronic $T_{2}$ . From physical considerations, $r_{1}\geq 1$ . The ratio $r_{2}$ relates the diffusion timescale τ to the electronic $T_{2}$ . Since $T_{2S}$ is rather long, we expect τ to be shorter and hence $r_{2}\geq 1$ . Finally, the ratio $r_{3}$ expresses the actual factor $N/3b^{3}$ , which is unknown, as a multiple of this same factor for arbitrarily selected reference values $N_{\mathrm{ref}}$ and $b_{\mathrm{ref}}$ . In principle, $r_{3}$ can be any positive number.

The mean volume per particle at a concentration of 1 M is 1.66 nm ${}^{3}$ and corresponds to a cube with side length of 1.18 nm. From molecular modeling, the “radius” of a BDPA molecule is about 0.6 nm, so it barely fits in the above cube. The partial molecular volume of a DMPC lipid in a lipid bilayer is 1.1 nm ${}^{3}$ [Bibr bib1.bibx17]. Thus, the concentration of one BDPA when surrounded by 10 DMPC lipids cannot exceed $N_{\mathrm{ref}}=0.1$ M but is also likely close to this value. Additionally taking $b_{\mathrm{ref}}=1$ nm in the last equality of Eq. ([Disp-formula Ch1.E87]), we anticipate $r_{3}>1$ .

From Eq. ([Disp-formula Ch1.E10]), the expected dependence of the DNP enhancement on the three fitting parameters can be written as

88 $$\epsilon_{\mathrm{SE}}\approx p(r_{1})v_{-}(r_{1},r_{2})r_{3}\langle \delta_{\mathrm{ref}}^{2}\rangle T_{1I}|\gamma_{S}|/\gamma_{I},$$

where $\langle \delta_{\mathrm{ref}}^{2}\rangle$ is calculated according to Eq. ([Disp-formula Ch1.E27]) using $N_{\mathrm{ref}}$ and $b_{\mathrm{ref}}$ . The ratio $r_{1}$ , which determines $\Omega_{1/2}$ (Eq. [Disp-formula Ch1.E82]), will influence the electronic polarization factor (or, equivalently, saturation factor). Together with the ratio $r_{2}$ , it will also influence the forbidden transition rate $v_{-}$ , although the effect of $r_{1}$ is expected to be small. From the previous discussion, we expect that $r_{2}$ will mostly be responsible for the width of the solid-effect lines that comprise $v_{-}$ . Finally, $r_{3}$ will serve as a global scaling factor that will adjust the amplitude of the overall enhancement. Since the three fitting parameters are responsible for different features of the DNP spectrum, it should be possible to determine them uniquely.

The top plot in Fig. [Fig Ch1.F7] shows the experimental enhancements (red circles) together with the best fit obtained using the FFHS model with $B_{1}=6$ G (solid black line). The solid green line in this plot is the difference between the experimental data and the fit. The corresponding fitting parameters are shown in the first row of Table [Table Ch1.T1]. Note that the fits were performed numerically using the exact expressions of p , $v_{\pm }$ , and the DNP enhancement (Eq. [Disp-formula Ch1.E9]) and were not restricted to the dependencies on the fitting parameters $r_{i}$ ( i=1,2,3 ) that are indicated in Eq. ([Disp-formula Ch1.E88]). (For example, the general dependence of the electronic polarization factor p on $T_{1S}$ and $T_{2S}$ is not limited to the ratio $T_{1S}/T_{2S}$ .)

After the fits converged, we used the final values of the fitting parameters to calculate the dispersive component of the EPR line $s_{x}^{\mathrm{ss}}=p\omega_{1}f_{x}\;s_{z}^{\mathrm{eq}}$ , and the factor $T_{x}$ , such that $(p\omega_{1}f_{x})T_{x}=pv_{-}$ . These are shown in the lower plot of Fig. [Fig Ch1.F7], where $s_{x}^{\mathrm{ss}}$ (blue) and $T_{x}$ (orange) are scaled independently along the vertical axis. Their product (dotted–dashed black line) is also scaled independently along the y axis. Since $p_{X}\approx 1$ in our case, the product $pv_{-}$ is itself proportional to the solid-effect enhancement. Hence the black lines in the upper and lower plots of Fig. [Fig Ch1.F7] are directly comparable. We can thus visually conclude that the unusual enhancement peaks at small offsets are a direct manifestation of the dispersive component of the power-broadened EPR line.

**Figure 7 Ch1.F7:**
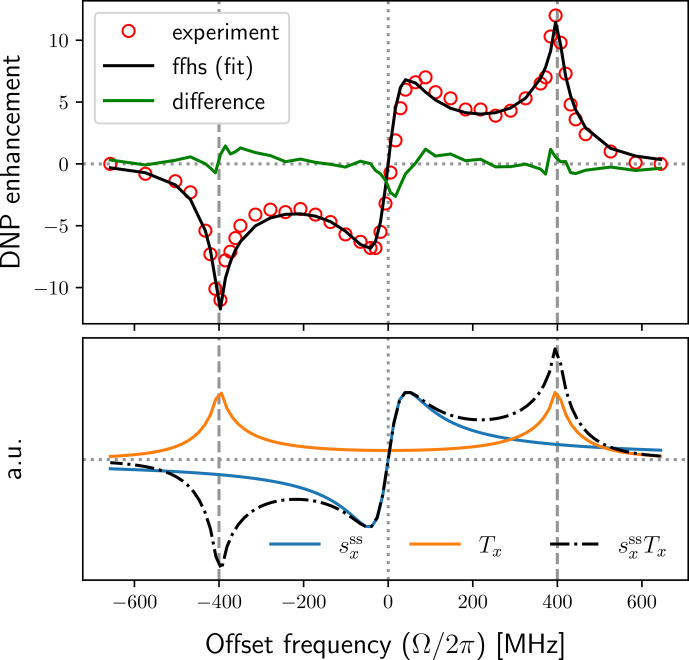
Experimental DNP field profile at J band (red circles) and fit with the FFHS model using $B_{1}=6$ G (solid black line). The central peaks in the enhancement profile follow the dispersive component of the power-broadened EPR line (solid blue line).

**Table 1 Ch1.T1:** Fitting parameters $r_{i}$ ( i=1,2,3 ) and implied timescales ( $T_{1S}$ and τ ) and contact distance ( b ). For different mw fields, $r_{1}$ changes such that $B_{1}\sqrt{r_{1}}$ remains constant.

$B_{1}$ (G)	$r_{1}$	$r_{2}$	$r_{3}$	$T_{1S}$ ( µ s)	τ (ns ${}^{-1}$ )	b (nm ${}^{*}$ )	$B_{1}\sqrt{r_{1}}$
6	7.2	30.4	8.9	1.5	7.07	0.482	16.088
5	10	30.4	13	2.2	7.08	0.427	16.065
4	16	30.3	20	3.5	7.09	0.368	16.047
3	29	30.3	36	6.1	7.10	0.304	16.034
2	64	30.3	80	14	7.10	0.232	16.025
1	257	30.3	321	55	7.11	0.146	16.022

${}^{*}$ Assuming radical concentration N=0.1 M.

Because the mw field in the experiment is not known precisely, we also attempted fits with smaller $B_{1}$ . The best fits were practically identical to the one shown in Fig. [Fig Ch1.F7] but with different values of the fitting parameters (Table [Table Ch1.T1]). (The fit for $B_{1}=2$ G is shown in Fig. [Fig App1.Ch1.S1.F9] as an example.)

From Table [Table Ch1.T1] we see that all fits resulted in the same value of the parameter $r_{2}$ , implying τ=7.1 ns for the motional timescale of the FFHS model. This parameter is very robust because it directly reflects the width of the experimental solid-effect lines at $\Omega =\pm \omega_{I}$ . Although, normally, their line width should depend on both $T_{2S}$ and τ , the exceptionally narrow EPR line puts us in the regime $\tau \ll T_{2S}$ where the influence of $T_{2S}$ is negligible. As a result, the motional broadening of the solid-effect lines in the DNP spectrum reports directly on the diffusive timescale of BDPA in the lipid environment.

The fitting parameter $r_{1}$ adjusts the extrema of the dispersive EPR line (solid blue line in Fig. [Fig Ch1.F7]), which are at $\Omega_{1/2}=\pm \omega_{1}\sqrt{r_{1}}$ (Eq. [Disp-formula Ch1.E82]). By monitoring the product of $\sqrt{r_{1}}$ and $B_{1}$ in the last column of Table [Table Ch1.T1], we see that, each time $B_{1}$ is modified, the fitted $r_{1}$ changes such that $\Omega_{1/2}$ remains unchanged, as required by the positions of the non-canonical enhancement peaks in the experimental data. However, because $r_{1}$ has to increase quadratically to compensate for the reduction of $B_{1}$ , the implied electronic $T_{1}$ times become exceedingly long (tens of microseconds) at the smaller values of $B_{1}$ (1–2 G).

When $B_{1}$ is reduced, the fitting parameter $r_{3}$ also increases quadratically to compensate for the dependence of the overall enhancement on $\omega_{1}^{2}$ . Assuming $N_{\mathrm{ref}}=0.1$ M is a good estimate of the actual concentration of BDPA in the lipid bilayer, it is possible to calculate a contact distance, b , from the fitted value of $r_{3}$ . The deduced contact distances are given in the second last column of Table [Table Ch1.T1]. Only the values for large $B_{1}$ (5–6 G) are in qualitative agreement with the molecular structure of BDPA.

**Figure 8 Ch1.F8:**
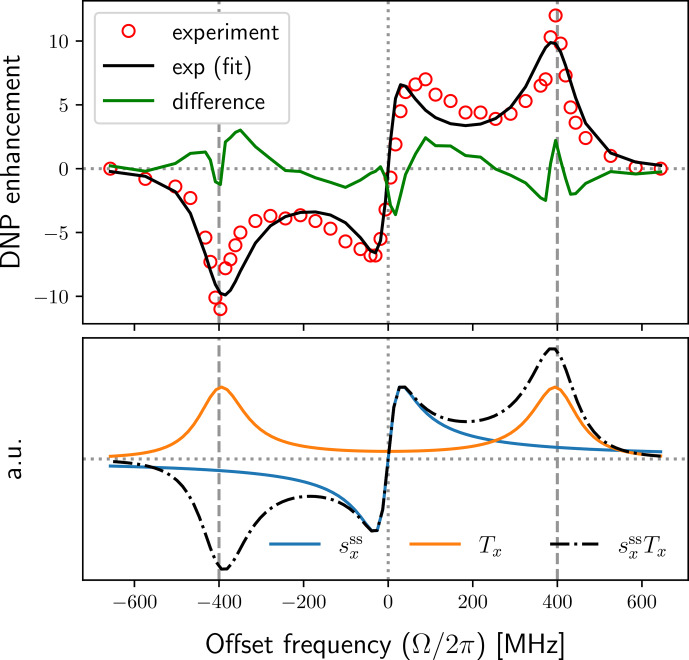
Same as Fig. [Fig Ch1.F7] for the model with mono-exponential dipolar correlation function and $B_{1}=6$ G (solid black line). The fit parameters $r_{1}=3.6$ , $r_{2}=89$ and $r_{3}=7.7$ correspond to $T_{1S}=0.77\;µ$ s, τ=2.4 ns and b=0.507 nm (assuming N=0.1 M).

In Fig. [Fig Ch1.F8] we show a fit to the same experimental data using a mono-exponential dipolar correlation function. While the difference between the data and the fit (green line in top panel of Fig. [Fig Ch1.F8]) is not much worse than what we had for the FFHS model, it is apparent that the exponential model strives to find the right balance between the broadening of the solid-effect lines and the tails of these lines at the lower offsets, ultimately producing too broad solid-effect lines and too narrow non-canonical peaks. (An exponential fit with $B_{1}=4$ G is shown in Fig. [Fig App1.Ch1.S1.F10].) We thus see that the J-band DNP spectrum clearly differentiates between two alternative motional models, ruling out the less realistic one.

The most certain outcome of the fits with the FFHS model is the deduced motional timescale τ , as it comes directly from the width of the solid-effect lines ( $T_{2S}$ is too long to contribute). The deduced value of $T_{1S}$ is somewhat less certain since it is accessed relative to $T_{2S}$ and also depends on the mw field $B_{1}$ . Nevertheless, with reasonable choices of $T_{2S}$ and $B_{1}$ , the fit to the non-canonical extrema in the DNP spectrum restricts $T_{1S}$ to a meaningful window between 1.5 and 2.5 µ s (Table [Table Ch1.T1]). Least certain is the estimate of the contact distance b since, in addition to $B_{1}$ , it requires precise knowledge of the radical concentration and the nuclear spin-lattice relaxation time. Although the latter is accessible experimentally, its value was measured at 298 K, while the DNP measurements are at 320 K.

In spite of the uncertainty in the estimated value of the distance parameter b , let us use τ and b in Eq. ([Disp-formula Ch1.E67]) to calculate the coefficient of relative translational diffusion. With b=0.482 nm (Table [Table Ch1.T1], first row) and τ=7.1 ns, we get $D=b^{2}/\tau =0.033\;{\mathrm{nm}}^{2}\;{\mathrm{ns}}^{-1}=33\times 10^{-12}\;m^{2}\;s^{-1}$ . Alternatively, with b=0.427 nm (Table [Table Ch1.T1], second row) and τ=7.1 ns, we find $D=26\times 10^{-12}\;m^{2}\;s^{-1}$ . The first value corresponds to $B_{1}=6$ G and the second to $B_{1}=5$ G.

For comparison, the coefficient of lateral diffusion of phospholipids in oriented DMPC bilayers, as determined from pulsed field gradient NMR, is about $11\times 10^{-12}\;m^{2}\;s^{-1}$ at 308 K, $20\times 10^{-12}\;m^{2}\;s^{-1}$ at 323 K and $27\times 10^{-12}\;m^{2}\;s^{-1}$ at 333 K Fig. 5b, 0 mol % cholesterol. As the temperature of the DNP measurements is closer to the middle value, our two estimates of D are seen to be larger by a factor of 1.65 and 1.3, respectively.

However, the D of the FFHS model corresponds to the relative translational diffusion of the electronic and nuclear spins, i.e., $D=D_{S}+D_{I}$ , where $D_{S}$ and $D_{I}$ denote the coefficients of translational diffusion of the two spin types. Disregarding all complicating factors, one could thus take $D_{I}=20\times 10^{-12}\;m^{2}\;s^{-1}$ from the literature value and rationalize the values that we deduced from the width of the solid-effect DNP lines as implying either $D_{S}=13\times 10^{-12}\;m^{2}\;s^{-1}$ or $D_{S}=6\times 10^{-12}\;m^{2}\;s^{-1}$ for the diffusion coefficient of the free radical BDPA in the lipid bilayer. As the obtained numerical values are rather plausible, we conclude that the quantitative analysis of the J-band DNP spectrum leads to meaningful molecular properties. Without the theoretical framework developed in this paper, neither the molecular distance b nor the diffusion coefficient D would be accessible from a solid-effect DNP spectrum in the liquid state.

## 5 Concluding discussion

Erb, Motchane and Uebersfeld had the hunch that the dispersive component of the EPR line is reflected in the solid-effect DNP enhancement [Bibr bib1.bibx8]. A theoretical justification of their intuition was provided in the companion paper [Bibr bib1.bibx37]. Here, the formalism was extended to the solid effect in liquids. Our theoretical predictions were compared with recent DNP measurements at high field [Bibr bib1.bibx27]. The comparison demonstrated that, under appropriate conditions, the dispersive component of the EPR line is literally visible in the field profile of the DNP enhancement. Provided that seeing is believing, we have thus closed the circle.

The DNP mechanism which became known as the solid-state effect due to Abragam [Bibr bib1.bibx2] had been observed in liquids from the very beginning [Bibr bib1.bibx8]. Nevertheless, perhaps because it yielded comparatively smaller absolute enhancements and often coexisted with the Overhauser effect [Bibr bib1.bibx29], the solid effect has remained less explored in liquids compared to solids. The recent use of this DNP mechanism as a new modality for probing the molecular dynamics in ionic liquids [Bibr bib1.bibx31] and its first applications at high magnetic field [Bibr bib1.bibx27] indicate that the potential of the solid effect in the liquid state is yet to be harvested. A theoretical understanding of the mechanism in liquids is clearly going to be helpful in these endeavors. Developing the needed theory has been the main aim of the companion and current papers.

Admittedly, a theoretical description of the solid effect in liquids was developed more than 50 years ago by Korringa and colleagues [Bibr bib1.bibx34]. In fact, their analysis was much more ambitious than ours, as it aimed to quantify the DNP spectrum during the transition from the Overhauser effect to the solid effect upon reduction of the experimental temperature [Bibr bib1.bibx29]. Thus, in addition to the secular terms of the dipolar interaction that we considered here and in [Bibr bib1.bibx37], their Hamiltonian also contained the non-secular terms, which are important for the cross-relaxation rates of the Overhauser effect, as well as the orientation-dependent part of the electronic Zeeman interaction, which determines the electronic relaxation rates and thus the degree of saturation. Following the prescription of second-order time-dependent perturbation theory, Korringa et al. derived equations for the deviations of both the electronic and nuclear polarizations from their values at thermal equilibrium [Bibr bib1.bibx34].

The analytical framework of Korringa and colleagues had two additional aspects. First, as is well known, the semi-classical description of spin-lattice relaxation relaxes the system to infinite temperature. The usual way of correcting for this shortcoming in magnetic resonance is to subtract the correct thermal equilibrium from the right-hand side of the dynamical equation of the density matrix [Bibr bib1.bibx1]. Instead, [Bibr bib1.bibx22] imposed the correct temperature by writing the equation of motion of the density matrix for complex-valued time, whose imaginary part was proportional to the inverse temperature. This mathematical trick exploits the fact that a quantum-mechanical propagator with imaginary time becomes a Boltzmann factor. The analytical continuation to complex time modified the familiar Liouville–von Neumann equation of the density matrix to a form that is not common in magnetic resonance. Second, as an integral part of their formalism, [Bibr bib1.bibx23] modeled the stochastic modulation of the spin Hamiltonian as rotational diffusion of one coordinate frame with respect to another, which led to an exponential correlation function with single decay time τ . It is not straightforward to see how their final analytical expressions should be modified if one were to use the FFHS model, for example.

In this context, it is worth mentioning that the mono-exponential model did not accurately fit the experimental data of [Bibr bib1.bibx29], and the authors took a Gaussian distribution for ln⁡τ [Bibr bib1.bibx28]. In Fig. [Fig Ch1.F8] we also observed that an exponential motional model did not fit the experimental DNP spectrum at J band [Bibr bib1.bibx27], whereas the FFHS model with a single motional parameter did (Fig. [Fig Ch1.F7]). One should remember, however, that the analysis of [Bibr bib1.bibx29] was performed 4 years before the spectral density of the FFHS model was solved analytically [Bibr bib1.bibx4].

In spite of the differences between the analytical framework of Korringa and colleagues [Bibr bib1.bibx34] and our approach, which hamper a direct comparison of the results, we observe that the derivations in their first paper [Bibr bib1.bibx34] assumed isotropic electronic relaxation, i.e., $T_{1S}=T_{2S}$ . As we saw in Sect. [Sec Ch1.S4.SS2], in this case the matrix B (Eq. [Disp-formula Ch1.E13]) becomes equal to $B_{0}$ (Eq. [Disp-formula Ch1.E69]) and the eigenvalue problem of the latter has a simple closed-form solution. All quantities of interest then become linear combinations of the reciprocals of the eigenvalues. Indeed, the final expressions of [Bibr bib1.bibx34] are linear combinations of Lorentzian spectral densities, which contain the effective frequency $\omega_{\mathrm{eff}}$ (Eq. [Disp-formula Ch1.E71]).

The assumption of equal longitudinal and transverse electronic relaxation rates was relaxed in the second paper [Bibr bib1.bibx28]. Sadly, this second paper has been cited only five times, and although all of the citing papers report new experiments, they do not use the theoretical expressions of [Bibr bib1.bibx28] to analyze the experimental data. One can only hope that, by being less ambitious, the theory developed in the current paper fares differently.

## Figures and Tables

**Figure A1 App1.Ch1.S1.F9:**
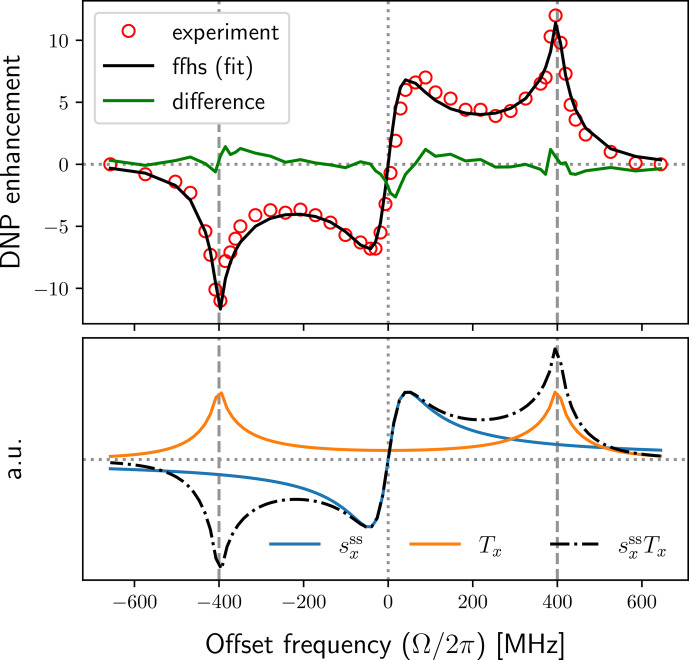
Same as Fig. [Fig Ch1.F7] (FFHS model) for $B_{1}=2$ G (solid black line). The fit parameters are given in the second last row of Table [Table Ch1.T1].

**Figure A2 App1.Ch1.S1.F10:**
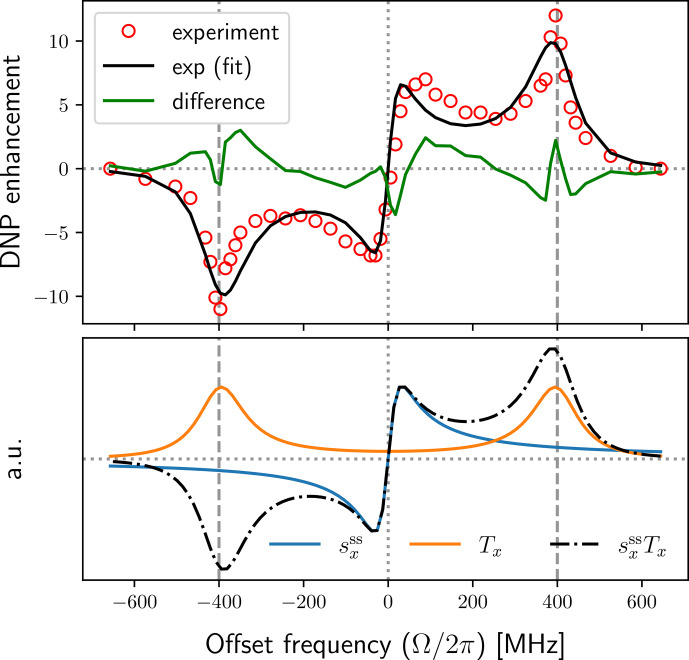
Same as Fig. [Fig Ch1.F8] (exponential model) for $B_{1}=4$ G (solid black line). The fit parameters $r_{1}=8.1$ , $r_{2}=90$ , $r_{3}=17$ correspond to $T_{1S}=1.7\;µ$ s, τ=2.4 ns, b=0.387 nm (assuming N=0.1 M).

## Data Availability

The analyzed data are at https://github.com/dzsezer/solidDNPliquids/data (https://doi.org/10.5281/zenodo.7990757, Sezer, 2023b).

## Code availability

The code used to generate the figures is at https://github.com/dzsezer/solidDNPliquids (https://doi.org/10.5281/zenodo.7990757, Sezer, 2023b).
